# A comprehensive enhancer screen identifies TRAM2 as a key and novel mediator of YAP oncogenesis

**DOI:** 10.1186/s13059-021-02272-8

**Published:** 2021-01-29

**Authors:** Li Li, Alejandro P. Ugalde, Colinda L. G. J. Scheele, Sebastian M. Dieter, Remco Nagel, Jin Ma, Abhijeet Pataskar, Gozde Korkmaz, Ran Elkon, Miao-Ping Chien, Li You, Pin-Rui Su, Onno B. Bleijerveld, Maarten Altelaar, Lyubomir Momchev, Zohar Manber, Ruiqi Han, Pieter C. van Breugel, Rui Lopes, Peter ten Dijke, Jacco van Rheenen, Reuven Agami

**Affiliations:** 1 Division of Oncogenomics, The Netherlands Cancer Institute, Plesmanlaan 121, 1066CX Amsterdam, The Netherlands; 2 Division of Molecular Pathology, The Netherlands Cancer Institute, Plesmanlaan 121, 1066 CX Amsterdam, The Netherlands; 3 Department of Molecular Cell Biology, Cancer Genomics Centre Netherlands, Leiden University Medical Center, Leiden, The Netherlands; 4 Department of Human Molecular Genetics and Biochemistry, Sackler School of Medicine, Tel Aviv University, Tel Aviv, Israel; 5 Department of Molecular Genetics, Erasmus University Medical Center, Rotterdam, The Netherlands; 6 Proteomics Facility, The Netherlands Cancer Institute, Plesmanlaan 121, 1066 CX Amsterdam, The Netherlands; 7 Biomolecular Mass Spectrometry and Proteomics, Bijvt Centre for Biomolecular Research and Utrecht Institute for Pharmaceutical Sciences, University of Utrecht, Padualaan 8, 3584CH Utrecht, The Netherlands; 8 Erasmus MC, Rotterdam University, Rotterdam, The Netherlands

**Keywords:** YAP, Enhancer, TRAM2, Proliferation, Migration, Invasion, Gene regulation

## Abstract

**Background:**

Frequent activation of the co-transcriptional factor YAP is observed in a large number of solid tumors. Activated YAP associates with enhancer loci via TEAD4-DNA-binding protein and stimulates cancer aggressiveness. Although thousands of YAP/TEAD4 binding-sites are annotated, their functional importance is unknown. Here, we aim at further identification of enhancer elements that are required for YAP functions.

**Results:**

We first apply genome-wide ChIP profiling of YAP to systematically identify enhancers that are bound by YAP/TEAD4. Next, we implement a genetic approach to uncover functions of YAP/TEAD4-associated enhancers, demonstrate its robustness, and use it to reveal a network of enhancers required for YAP-mediated proliferation. We focus on Enhancer^TRAM2^, as its target gene TRAM2 shows the strongest expression-correlation with YAP activity in nearly all tumor types. Interestingly, TRAM2 phenocopies the YAP-induced cell proliferation, migration, and invasion phenotypes and correlates with poor patient survival. Mechanistically, we identify FSTL-1 as a major direct client of TRAM2 that is involved in these phenotypes. Thus, TRAM2 is a key novel mediator of YAP-induced oncogenic proliferation and cellular invasiveness.

**Conclusions:**

YAP is a transcription co-factor that binds to thousands of enhancer loci and stimulates tumor aggressiveness. Using unbiased functional approaches, we dissect YAP enhancer network and characterize TRAM2 as a novel mediator of cellular proliferation, migration, and invasion. Our findings elucidate how YAP induces cancer aggressiveness and may assist diagnosis of cancer metastasis.

**Supplementary Information:**

The online version contains supplementary material available at 10.1186/s13059-021-02272-8.

## Background

The YAP (YES-associated protein) and its paralog, the transcriptional coactivator with PDZ-binding motif (TAZ), have attracted increasing attention in the last decade for their ability to stimulate cell proliferation and their frequent activation in a large number of solid tumors [1]. These two highly related co-transcription factors function downstream of the Hippo pathway, an evolutionarily conserved signaling network that senses cell polarity and regulates adhesion, cell death, and differentiation [2, 3]. The Hippo pathway is an serine/threonine kinase signaling cascade originally identified in fruit fly [4, 5] that is known for its involvement in controlling organ size and development [6, 7]. In mammals, the Hippo pathway negatively regulates the activity of the transcriptional co-activators YAP and TAZ. Phosphorylation of YAP and TAZ by the large tumor suppressor 1 and 2 kinases (LATS1 and LATS2) either gives rise to their association with the 14-3-3 proteins leading to cytoplasmic sequestration of YAP/TAZ [8, 9] or to ubiquitin-mediated protein degradation [10].

Accumulating evidence indicate that YAP/TAZ control key determinants of cancer biology, including cell proliferation and chemotherapeutic drug resistance, and have significant impact on patient prognosis [11–14]. For example, liver-specific YAP overexpression in mouse models caused hepatocellular carcinoma [15]. Activated YAP was also demonstrated to mediate tumor progression, metastasis, and drug resistance in lung cancer cells [16–19]. Last but not the least, high expression of YAP/TAZ, as well as their increased nuclear localization, was shown to correlate with poor prognosis in many cancer types [20–23]. Thus, YAP/TAZ are tightly linked to tumor development and aggressiveness.

YAP/TAZ appear as a significant central node in cellular signaling. Their activity is triggered by a wide-variety of cell-intrinsic and extrinsic cues, such as cell density, cell polarity, mechanical stress, and ligands of G protein-coupled receptors (GPCRs), as well as changes in cellular energy related to glucose and lipid metabolism [8, 24–27]. Mechanistically, unphosphorylated YAP/TAZ are able to translocate to the nucleus, where they activate gene expression programs by interacting with DNA-binding transcription factors, most prominently from the TEA domain gene family (TEAD) [28]. While it has been initially reported that YAP regulates the expression of numerous target genes through binding to promoters, recent studies have demonstrated that YAP modulates gene transcription through binding to thousands of distal transcriptional enhancer regions [29–32].

Transcriptional enhancers are functional regulatory DNA elements that activate the expression of their distant target genes [33]. These elements are highly abundant in mammalian genomes (in the range of hundreds of thousands), and they are currently viewed as the main determinants of the spatiotemporal regulation of gene expression. Enhancers can be predicted indirectly by specific chromatin marks, most notably high H3K4me1 and H3K27ac, and low H3K4me3 [34]. Mechanistically, enhancers serve as binding platform for transcription factors that regulate target gene transcription through chromatin loops. Importantly, the binding of transcription factors to their target enhancers, however, is not always the direct evidence for enhancer function [35], and the expression of the so-called enhancer-associated RNA as a result of their activation better correlates with their activity [36, 37].

The function of the vast majority of the enhancers is poorly understood, and the lack of specific knowledge on the rules that govern the interaction between enhancers and their regulated target genes prevents the prediction of their contribution to phenotypes. However, recent publications using CRISPR-Cas9-mediated functional genetic screening approaches paved the way to experimentally dissect key roles of transcriptional enhancer networks [38, 39].

To uncover key enhancer functions of YAP, we combined a genome-wide and robust CRISPR-Cas9-mediated approach for YAP activity by targeting YAP/TEAD4-bound enhancers in MCF10A cells with activated YAP overexpression. This assay has identified a handful of YAP-regulated enhancers required for cellular proliferation. Beyond the known targets of YAP (i.e., MYC and CCND1), we uncovered TRAM2 (translocation chain-associated membrane 2) and described its contribution to cellular proliferation, epithelial to mesenchymal transition (EMT), and cellular migration and invasion, as well as cancer prognosis. TRAM2 is a component of the translocon, a gated channel that controls the posttranslational processing of nascent secretory and membrane proteins at the endoplasmic reticulum (ER) membrane [40–42]. We have found that FSTL-1 is a major client of TRAM2 involved in the YAP-induced, TRAM2-dependent phenotypes. Collectively, our findings do not only detail a new regulatory mode-of-function for YAP in tumorigenesis, but also demonstrate the benefits in performing functional enhancer screens by silencing specific transcriptional activation nodes without affecting the basal expression and potential essentiality of their target genes.

## Results

### A functional genetic screen for activated YAP-mediated deregulated proliferation

To setup a functional genetic screen for YAP oncogenic activity, we generated a cellular system based on the non-transformed mammary epithelial cell line MCF10A. We transduced these cells with constitutively active and inactive YAP variants (YAP^5SA^ and YAP^5SA-S94A^, respectively) and control empty vector and examined their morphology and capacity to form mammospheres. In line with a previous report [29], the expression of the constitutively active form of YAP (YAP^5SA^, named here activated YAP), but not the inactive variant (YAP^5SA-S94A^, YAP^S94A^ mutation disrupts YAP-TEAD interaction), changed the epithelial cellular morphology of MCF10A to resemble a mesenchymal phenotype (EMT) and provoked a transformed phenotype manifested in clonogenic outgrowth (Additional file 1: Fig. S1A). The mild overexpression of YAP^5SA^ and YAP^5SA-S94A^ was confirmed by immunoblot (Additional file 1: Fig. S1A). Importantly, activated YAP also induced deregulated proliferation (Additional file 1: Fig. S1B), in line with a previous report [43].

**Fig. 1:**
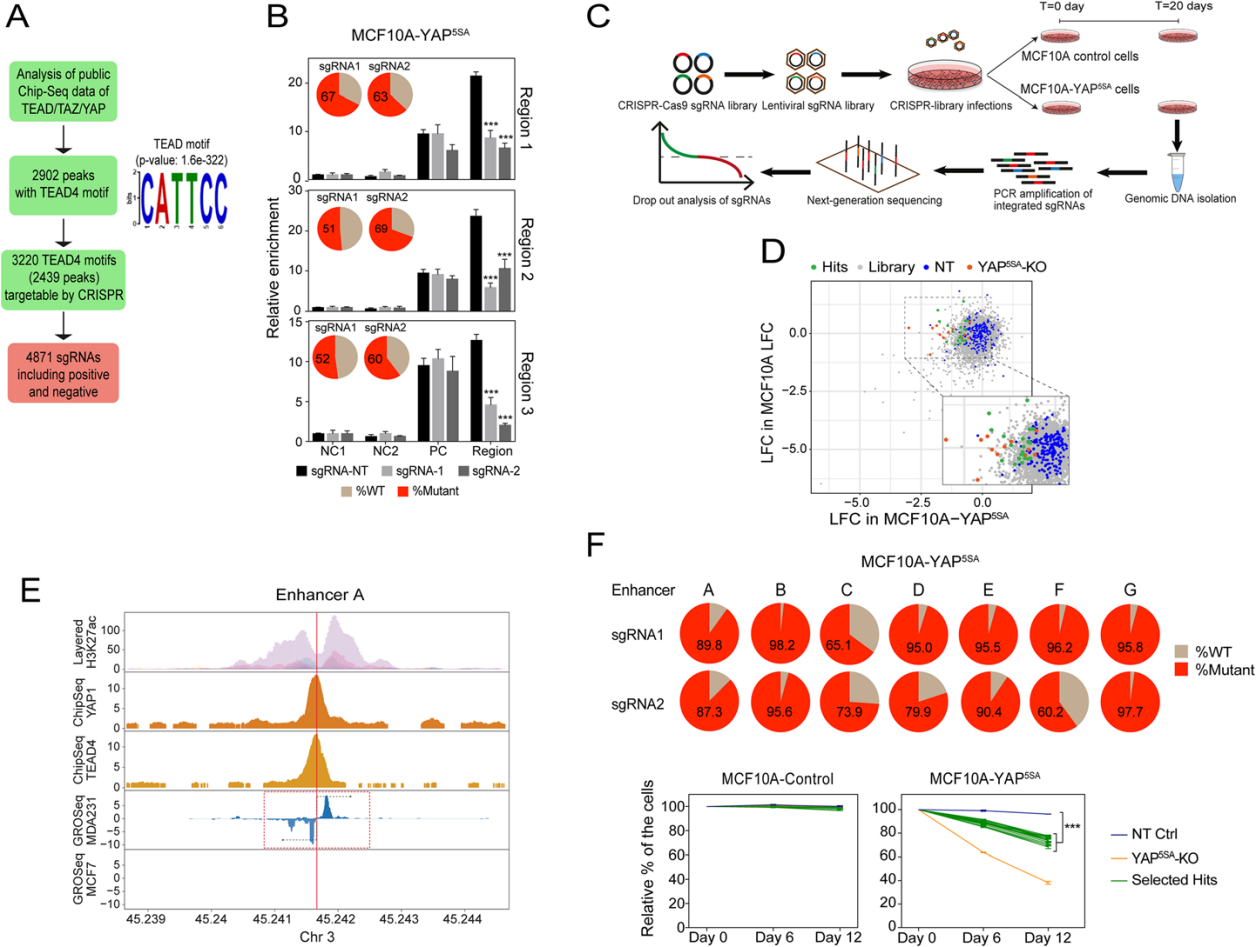
CRISPR-YAP-enh-lib library design, evaluation, and functional screening. **a** A schematic representation of the bioinformatics pipeline for the design of short guide RNAs (sgRNA) for CRISPR-YAP-enh-lib library construction. **b** ChIP-qPCR assays were performed to evaluate CRISPR efficiency in blocking YAP-binding to three selected top YAP-bound regions (Region1 chr19:41729486-41729491; Region2 chr22:41686880-41686885; Region3 chr1:19336594-19336599). Relative YAP binding was calculated and normalized over negative control (NC1 and NC2) regions and input. PC, positive control region - promoter of ANKRD1, as described in [29]. sgRNA-NT is a non-targeting control CRISPR vector. Error bars indicate standard deviation (SD) calculated from three technical replicates. ***P < 0.005, two-tailed Student’s *t* test. The pie plots are mutational profiles of MCF10A-YAP^5SA^ cells transduced with sgRNAs targeting the TEAD4 motif of the selected regions. **c** A schematic representation of the setup of the functional screen for YAP enhancers, as detailed in the material and method section “CRISPR library construction and analysis”. **d** Results of the functional screen of YAP enhancers. Scatter plot showing the average LFC (log fold change) of the sgRNA abundances between *T* = 20 and *T* = 0 in both MCF10A-YAP^5SA^ and MCF10A control cells (calculated from 3 biological replicates). Colored in orange are the sgRNAs targeting the YAP^5SA^ (positive controls), blue dots are the negative control sgRNAs, and the green dots are the selected hits showing reduced abundance only in MCF10A-YAP^5SA^ cells. **e** A representative plot for enhancer A, one of the selected hits, supplemented with genomic information of the targeting sgRNA sites, H3K27ac levels (an active enhancer marker), YAP and TEAD4 binding in MDA-MB-231 cells, and GRO-seq data of MDA-MB-231 and MCF7 cells. The black arrows indicate the production of enhancer RNA (eRNA). The information about the other selected hits is shown in Fig. S2. **f** Validation of the selected hits by competitive proliferation assays in MCF10A-YAP^5SA^ and MCF10A control cells. Pie plots indicate the mutation status of MCF10A-YAP^5SA^ cells transduced with the indicated sgRNAs. NT Ctrl is a non-targeting sgRNA control vector. YAP^5SA^-KO is an sgRNA vector targeting YAP^5SA^. Values from days 6 and 12 were normalized to day 0. Error bars indicate SD from three biological replicates. ***P < 0.005, two-tailed Student’s *t* test

As the most activated YAP/TAZ/TEAD4-bound chromatin regions are located within enhancers [29], we used an in silico approach to interrogate enhancers in chromatin immunoprecipitation sequencing (ChIPseq) data of YAP, TEAD4, and TAZ in MDA-MB-231, a triple-negative breast cancer cell line with known high YAP activity [29]. We identified 2902 predicted enhancer regions containing the TEAD4 motif (Fig. 1a). We then wished to evaluate the effectiveness of CRISPR-targeting and our enhancer screen approach in MCF10A-YAP^5SA^ cells (MCF10A cells with activated YAP overexpression). For that, we used six designed individual CRISPR vectors to target three TEAD4 motifs within the top YAP-bound regions (two sgRNA per motif) and transduced them into MCF10A-YAP^5SA^ cells (Additional file 1: Fig. S1C). Subsequently, we determined the mutational load inflicted by these vectors and its consequence to YAP-binding by deep sequencing and ChIP-qPCR, respectively. As anticipated, in all six cases, the introduction of the TEAD4-motif-targeting CRISPR vectors in MCF10A-YAP^5SA^ cells, but not the non-targeting control vector (sgRNA-NT), generated mutations with a frequency of more than 50% (Fig. 1b; Additional file 1: Fig. S1D). Most importantly, in all six cases, we also observed selective and effective YAP binding, which was markedly reduced (> 50%) following TEAD4-motif CRISPR targeting (Fig. 1b). These results indicate that CRISPR targeting of TEAD4 motifs is both robust and effective, with an estimated less than 20% false-negative rate for TEAD4 motifs that are targeted by 2 or more CRISPR vectors per region.

Ensured by an effective approach, we used YAP/TEAD4 binding information to construct a CRISPR-Cas9 vector library (CRISPR-YAP-enh-lib) consisting of 4871 vectors targeting 2439 regions with 3220 TEAD4 motifs out of the 2902 predicted enhancer regions and including positive controls (CRISPR vectors targeting activated YAP^5SA^) and non-targeting negative controls (CRISPR vectors with scramble sgRNAs) (Fig. 1a; Additional file 2: Table S1). We then executed a functional genetic screen as shown in Fig. 1c. We transduced MCF10A-control and MCF10A-YAP^5SA^ cells with CRISPR-YAP-enh-lib at a multiplicity of infection (MOI) of ~ 0.3 and drug-selected and cultured the cells for 20 more days. Genomic DNA was isolated from the transduced and drug-selected cells at the start of the experiment (*T* = 0) and at day 20 (*T* = 20). Samples were subjected to PCR-sequencing analysis to determine the abundance of each CRISPR vector. We then computed the changes in abundance between *T* = 20 and *T* = 0 for every CRISPR vector in each cell line (Additional file 1: Fig. S2A; Additional file 3: Table S2) and performed a differential abundance analysis to identify sgRNAs that affect MCF10A-YAP^5SA^, but not MCF10A-Control cells (Fig. 1d). We first confirmed the robustness of the screen with the vast majority of the negative control sgRNAs (blue dots) showing no significant change in abundance in both cell lines, while the majority of the YAP^5SA^-targeting sgRNAs (YAP^5SA^-knock out (KO), orange dots) were significantly depleted only in MCF10A-YAP^5SA^ cells. To identify the TEAD4-motifs with potential selective activity in MCF10A-YAP^5SA^ cells, we calculated the average differential abundance of T20 vs T0 of the 2 most depleted sgRNA vectors for each region from three biological replicates and compared the difference in both cell lines. Then, we selected the top ten TEAD4-motif hits (green dots) with selective depletion only in MCF10A-YAP^5SA^ cells. As expected, positive YAP^5SA^-targeting sgRNA vectors showed the strongest effect from all the selected hits (Additional file 4: Table S3). We then used Global Run-On sequencing (GRO-seq) datasets to filter out enhancer hits that did not show a significant increase in enhancer-associated RNA (eRNA) levels in MDA-MB-231 compared with MCF7 cells (possessing high and low YAP activity, respectively [44–46]) (Fig. 1e; Additional file 4: Table S3; Additional file 1: Fig. S2B). This procedure pinpointed 7 enhancer regions out of the top 10 selected candidates, which we subjected to a more detailed follow-up analysis.

To further confirm screen results, and the endogenous enhancer activity of the selected YAP-bound regions, we transduced MCF10A-YAP^5SA^ and MCF10A-Control cells with individual CRISPR vectors (two sgRNAs for each TEAD4-motif) and first measured mutational load. As expected, we observed high frequency of mutations (60–90%) induced by all targeting vectors (Fig. 1f; Additional file 1: Fig. S3A). Then, we performed GFP-based growth competition assays to assess the effect of each CRISPR vector on cellular proliferation. As expected, the YAP^5SA^-targeting vector suppressed the proliferation of MCF10A-YAP^5SA^ cells only, while the negative control vector neither affected MCF10A-YAP^5SA^ nor MCF10A-Control cells (Fig. 1f). Depletion of the ectopically expressed YAP^5SA^ was verified by western blot (Additional file 1: Fig. S3B). Interestingly, all the seven screen-selected hits significantly compromised the proliferation of only MCF10A-YAP^5SA^ cells, albeit with a lower potency compared with the effect of YAP^5SA^-targeting vectors (Fig. 1f). Altogether, these results suggest that YAP regulates cell proliferation via a network of at least 7 TEAD4-bound enhancers, each of which with a relatively moderate contribution.

### A network of genes links activated YAP enhancers to the regulation of cellular proliferation

**Fig. 2:**
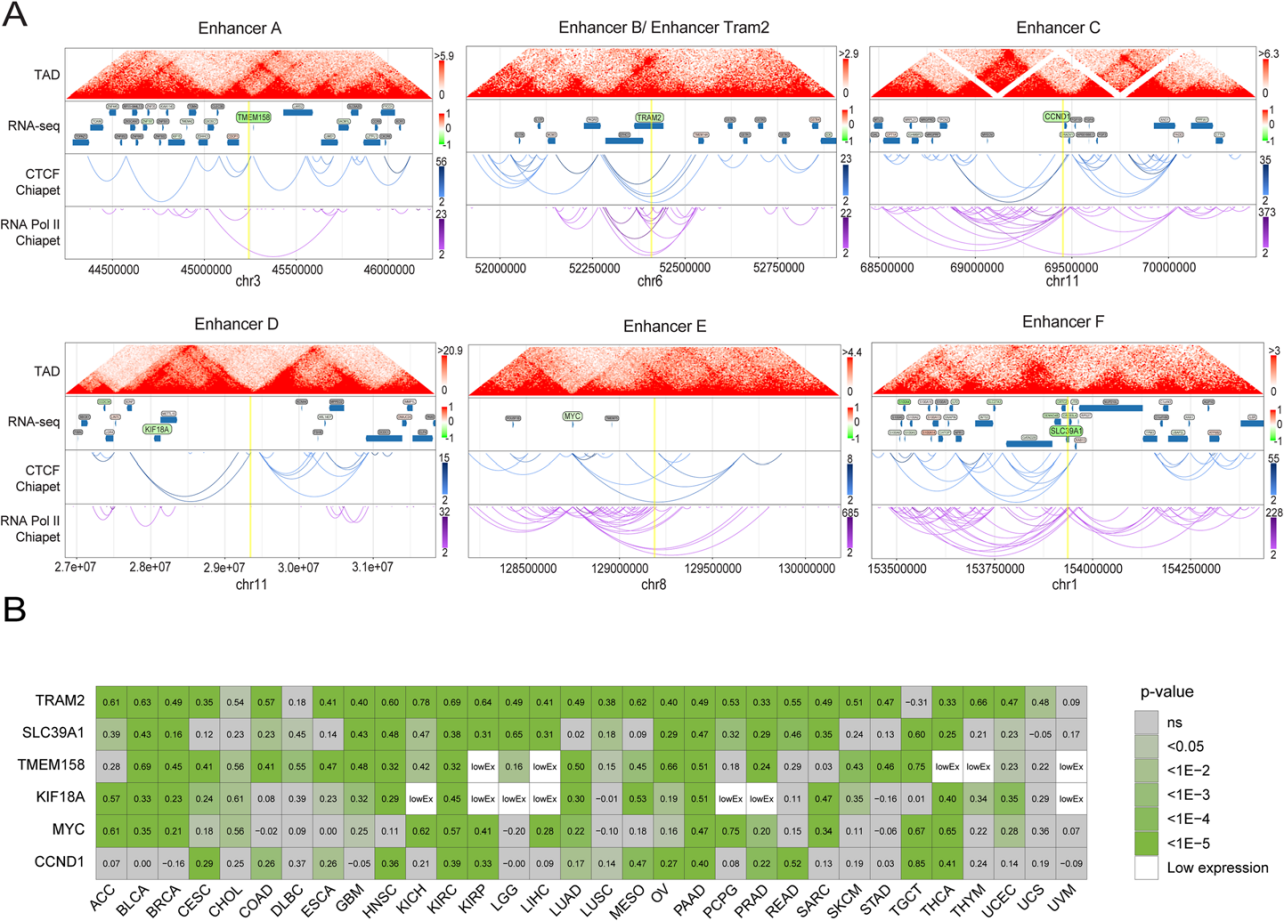
Target gene identification of the selected YAP enhancers. **a** Genomic views of the regions of the selected enhancers. The TAD (topologically associated domains) track represents a heatmap showing the frequency of chromatin interactions in 5 Kb bins in HMEC cells. The RNASeq/Ensembl track indicates known protein coding genes colored by the changes in gene expression (calculated as the average of log2 fold changes in gene expression between cells transduced with two different sgRNAs targeting the corresponding enhancer and non-targeting control sgRNAs). In gray are genes that did not significantly change. CTCF, ChIA-PET, and RNA Pol-II tracks were obtained from ENCODE from experiments performed with MCF7 cells using anti-CTCF and anti-RNA Pol-II, respectively (the color scales encode the number of reads supporting each interaction). **b** Heatmap plot showing the Pearson correlation coefficient between each selected enhancer target gene (**a**) and the YAP gene signature score in publicly available TCGA RNASeq samples from human tumors. The number in each tile represents the Pearson correlation coefficient in each TCGA study between the YAP gene signature score and the selected gene. The color scale represents the *p* value of correlation test, adjusted for multiple comparisons using Benjamini-Hochberg correction (ns, not significant; LowEx, low expression)

We sought to identify the critical functional target gene for each selected YAP enhancer. A major advantage of targeting enhancers as compared to gene bodies is that their disruption affects the induction but not the basal expression level of the target genes. In particular, this may have benefits in cases where target genes are essential. We therefore transduced MCF10A-YAP^5SA^ cells with individual sgRNA vectors targeting the 7 selected YAP enhancers (two validated sgRNA vectors per enhancer) and control (non-targeting (NT)). Subsequently, we performed mRNA sequencing and differential expression analysis to search for down regulated genes located within 1–5 Mbp from the sgRNA targeting sites (Fig. 2a; Additional file 1: Fig. S4A; Additional file 5: Table S4). This analysis uncovered potential targets for 6 out of the 7 selected YAP enhancers: TMEM158, TRAM2, CCND1, KIF18A, MYC, and SLC39A1. The location of every selected enhancer region and its corresponding target gene within the same topologically associating domain (TAD), as assessed by chromatin structure information from publicly available measurements, support direct genetic interactions (Fig. 2a).

Next, we interrogated the cancer genome atlas (TCGA) database to prioritize enhancer target genes by their expression correlation with a YAP gene signature score calculated from the expression of 57 genes defined in publication [47]. As anticipated, all selected target genes displayed significant correlation with YAP gene signature score in most of the tumor types (Fig. 2b), while nearby flanking genes showed a lower level of correlation for all enhancer regions (Additional file 1: Fig. S4B), reinforcing our enhancer target gene identification.

### YAP-mediated TRAM2 activation via Enhancer^TRAM2^ is required for YAP-mediated deregulation of cell proliferation

MYC and CCND1 are well-known targets of YAP [48, 49], and their contribution to cell proliferation, cell cycle, and cancer progression is well-established [50–56]. Nevertheless, we mapped here enhancer regions that wire YAP activation to the induction of these two genes. Furthermore, while a functional genetic interaction between YAP and TRAM2, KIF18A, TMEM158, and SLC39A1 was never reported, the role of TMEM158, KIF18A, and SLC39A1 in cell proliferation or cancer progression was already established before [57–62]. In contrast, no such information is available for TRAM2. TRAM2 is a translocating chain-associated membrane protein component of the translocon, a gated channel that controls the posttranslational processing of nascent secretory and membrane proteins at the endoplasmic reticulum (ER) membrane [40–42]. TRAM2 was shown to interact with SERCA2b, a Ca^2+^ pump, and that this interaction is required for TRAM2 function. However, TRAM2 was never linked to YAP, cellular proliferation, and human cancer.

**Fig. 3:**
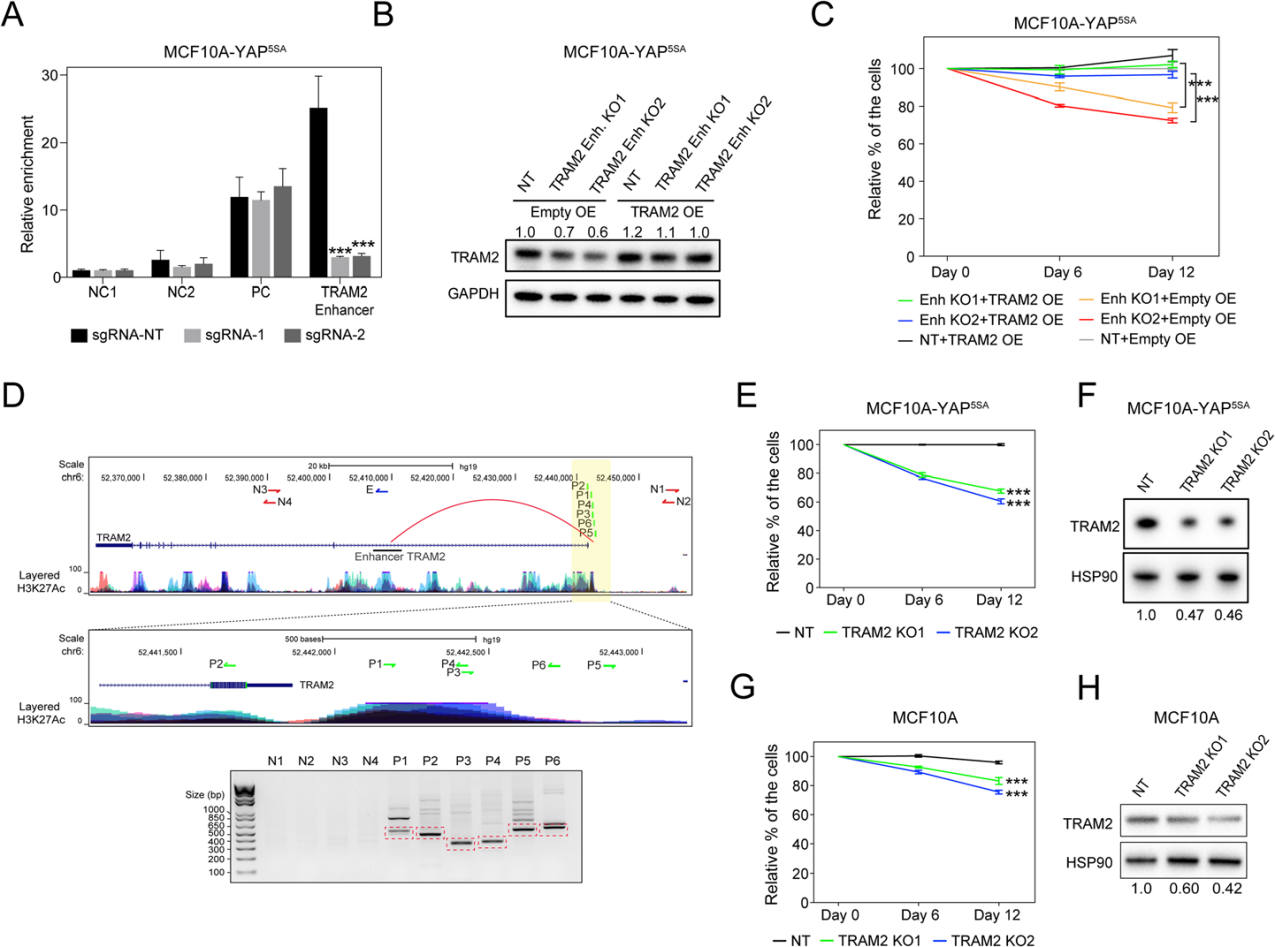
TRAM2 is a functional component of the YAP pathway. **a** ChIP-qPCR assays were used to quantify YAP-binding to Enhancer^TRAM2^ (chr6:52409174-52409286) in MCF10A-YAP^5SA^ cells transduced with two different sgRNAs (1 and 2) targeting the TEAD4 motif within Enhancer^TRAM2^. A non-targeting sgRNA (sgRNA-NT) was used as control. Relative YAP binding was calculated and normalized to NC1 and input (NC1, negative control region1; NC2, negative control region2; PC, positive control region). Error bars indicate SD from three biological replicates. ***P < 0.005, two-tailed Student’s *t* test. **b** Western blot analysis with anti-TRAM2 antibody using lysates obtained from MCF10A-YAP^5SA^ cells transduced with the indicated lentiviral vectors. NT, non-targeting sgRNA control. Anti-GAPDH antibody served as loading control. **c** Competitive proliferation of MCF10A-YAP^5SA^ cells transduced with the indicated lentiviral vectors. Values on days 6 and 12 were normalized to day 0. Error bars indicate SD from three biological replicates. ***P < 0.005, two-tailed Student’s *t* test. NT, non-targeting sgRNA control; TRAM2 overexpression (TRAM2 OE), and control vector (Empty OE). **d** 3C chromatin capture experiments were used to assess the interaction between Enhancer^TRAM2^ and the promoter of TRAM2. Genome browser representation of the location for each primer used in the 3C analysis. A constant primer E (blue arrow) was used to amplify Enhancer^TRAM2^. Control regions (N1–N4) were amplified with primers indicated in red arrows, and the TRAM2 promoter region was amplified with primers indicated in green arrows (P1–P6). An agarose gel image of the PCR products with the expected sizes (red frame). Sanger sequencing results from the indicated PCR products are shown in Fig. S5A. **e** Competitive proliferation assay, as described in **c**, of TRAM2-KOs in MCF10A-YAP^5SA^ cells. Values on day 6 (*T* = 6) and day 12 (*T* = 12) are normalized to day 0 (*T* = 0). Error bars indicate SD from three biological replicates. ***P < 0.005, two-tailed Student’s *t* test. **f** The cell populations used in **e** were subjected to a western blot analysis using anti-TRAM2 and control anti-HSP90 antibodies. **g** Competitive proliferation assay, as described in **c**, of TRAM2-KOs in MCF10A cells. Error bars indicate SD from three biological replicates. ***P < 0.005, two-tailed Student’s *t* test. **h** The cell populations used in **g** were subjected to a western blot analysis using anti-TRAM2 and control anti-HSP90 antibodies

We therefore sought to solidify the functional link between YAP, Enhancer^TRAM2^ (enhancer of TRAM2 gene), TRAM2, and cellular proliferation. We first validated that TRAM2 mRNA expression was downregulated upon Enhancer^TRAM2^-KOs (Additional file 1: Fig. S4C), which is in line with RNA-seq data (Fig. 2a; Additional file 5: Table S4). Then, we assessed the effect of CRISPR-targeting on the binding of YAP to Enhancer^TRAM2^ by ChIP-qPCR assay. Indeed, targeting the TEAD4 motif of Enhancer^TRAM2^ (Enhancer^TRAM2^-KO; two different sgRNAs) significantly reduced YAP binding to Enhancer^TRAM2^, but had no effect on a positive (PC) or two negative (NC) control regions (Fig. 3a). Western blot analyses confirmed the effect of Enhancer^TRAM2^-KO on TRAM2 protein expression (Fig. 3b). We therefore examined the ability of ectopically expressed TRAM2 (TRAM2 OE) in MCF10A-YAP^5SA^ cells to negate the phenotypic impact of targeting Enhancer^TRAM2^ on cell proliferation. Western blot analysis in MCF10A-YAP^5SA^ cells verified the mild ectopic expression of TRAM2 in control (NT) and Enhancer^TRAM2^-KO conditions compared to control (Empty OE) (Fig. 3b). This mild over-expression of TRAM2 was sufficient to compromise the reduction in the proliferation rate of MCF10A-YAP^5SA^-Enhancer^TRAM2^-KO cells (Fig. 3c).

We then postulated that if YAP mediates TRAM2 activation via Enhancer^TRAM2^, a physical interaction between TRAM2 promoter and the YAP-bound Enhancer^TRAM2^ should be expected. Indeed, chromatin conformation capture assays (3C) demonstrated a direct interaction between Enhancer^TRAM2^ (E) and TRAM2 promoter (P) (Fig. 3d). This genomic interaction is specific, as neighboring regions (N1-4) did not show any specific binding signal to Enhancer^TRAM2^ (Fig. 3d). The 3C PCR products were verified by Sanger sequencing (Additional file 1: Fig. S5A). We further observed a decrease in TRAM2 expression following verteporfin treatment (an inhibitor of the YAP-TEAD interaction) of MCF10A-YAP^5SA^ and MDA-MB-231 cells (Additional file 1: Fig. S5B). This indicates that YAP-TEAD association mediates TRAM2 activation via Enhancer^TRAM2^.

Lastly, we corroborated the role of TRAM2 in cell proliferation. CRISPR-mediated targeting of TRAM2 indicated an approximately 50% TRAM2 knockout by DNA mutation and western blotting analyses and a clear inhibitory effect on proliferation in both MCF10A-YAP^5SA^ and MCF10A cells (Fig. 3e–h; Additional file 1: Fig. S5C). Similar proliferation effect of Enhancer^TRAM2^-KO and TRAM2-KO was obtained in a second YAP-activated cellular system (MDA-MB-231, Additional file 1: Fig. S5D). Thus, TRAM2 is the prime functional target of YAP-mediated proliferation through Enhancer^TRAM2^.

### TRAM2 regulates EMT with increased cell migration and invasion but is insufficient to induce tumor formation

**Fig. 4:**
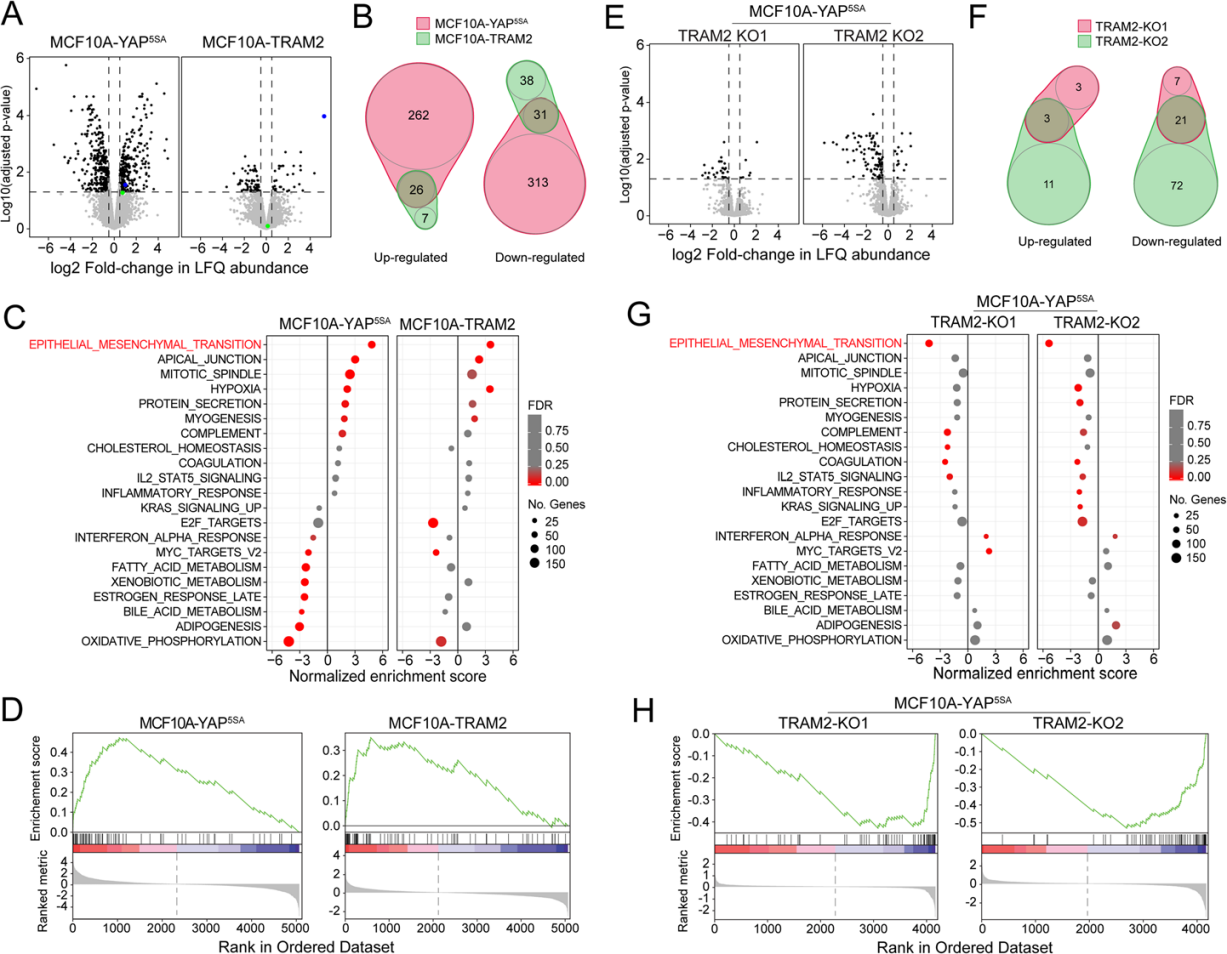
Proteomics analysis suggests a role for TRAM2 in YAP-induced EMT. **a** Volcano plots of the proteomics studies in MCF10A cells transduced with the indicated vectors. The data was compared and normalized to control cells. Dark dots are the proteins that were significantly upregulated or downregulated compered to control using a cutoff of absolute LFC > 0.5 and adjusted *p* value < 0.05. YAP is labeled green and TRAM2 blue. **b** Venn diagrams representing the significant overlap of upregulated (Fisher’s exact test *p* value 4.09E−27) or downregulated (Fisher’s exact test *p* value 6.11E−19) proteins between YAP and TRAM2 samples compared with control in **a**. **c** Bubble plot summarizing gene set enrichment analysis from the proteomics data presented in **a**. Significantly enriched gene sets (FDR < 0.25) in at least one condition were selected, and the normalized enriched scores were plotted for both samples. Bubble sizes represent the number of genes in each gene set, while the fill colors represent the FDR values as indicated. **d** GSEA plots of the EMT-hallmark molecular signature using the proteomics data described in **a**. **e** Volcano plots of proteomics studies in MCF10A-YAP^5SA^ cells transduced with the indicated vectors. The data was compared and normalized to control cells (non-targeting sgRNA). Dark dots are the proteins that were significantly upregulated or downregulated compered to control with cutoff of absolute LFC > 0.5 and adjusted *p* value < 0.05. **f** Venn diagrams representing the overlap of upregulated (Fisher’s exact test *p* value 6.00E−07) or downregulated (Fisher’s exact test *p* value 2.01E−30) proteins between TRAM2-KO1 and TRAM2-KO2 samples compared with control samples in the proteomics studies presented in **e**. **g** Bubble plot represents a gene set enrichment analysis from the proteomics data presented in **e**. Significantly enriched gene sets (FDR < 0.25) in at least one condition were selected, and the normalized enriched scores were plotted for both samples. Bubble sizes represent the number of genes in each gene set, while the fill colors represent the FDR values as indicated. **h** GSEA plots of the EMT-hallmark molecular signature using the proteomics data described in **e**

Since TRAM2 expression showed significant high correlation with the YAP gene signature score in almost all tumor types (Fig. 2b), and TRAM2 is a component of the translocon at the endoplasmic reticulum (ER), we next decided to investigate changes in protein expression between MCF10A-TRAM2 (MCF10A cells ectopically expressing TRAM2), MCF10A-YAP^5SA^ (MCF10A cells ectopically expressing activated YAP), and MF10A-Control (MCF10A cells ectopically expressing inactivated YAP,YAP^5SA-S94A^) cells using mass spectrometry (Fig. 4a, b; Additional file 6: Table S5). As expected, we observed elevated levels of TRAM2 (blue dot) in MCF10A-YAP^5SA^ and MCF10A-TRAM2 cells and mild YAP overexpression levels (green dot) only in MCF10A-YAP^5SA^ cells (Fig. 4a). Globally, we found hundreds of significantly and differentially expressed proteins (adjusted *p* value < 0.05 and |log2 fold-change| > 0.5) (288 upregulated and 344 downregulated) in MCF10A-YAP^5SA^ cells compared with control cells, as expected from YAP function as a master regulator of transcription (Fig. 4a, b; Additional file 6: Table S5). In contrast, MCF10A-TRAM2 cells induced a much milder global change (33 upregulated and 69 downregulated, Fig. 4a, b). Interestingly, 26 upregulated and 31 downregulated proteins were shared by both MCF10A-YAP^5SA^ and MCF10A-TRAM2 cells, indicating a significant overlap (Fisher exact test *p* value of 4.09E−27 and 6.11E−19, respectively; Fig. 4b). We therefore performed a gene set enrichment analysis (GSEA), which surprisingly indicated that the strongest enriched category of genes was linked with an EMT signature in both MCF10-YAP^5SA^ and MCF10A-TRAM2 cells (Fig. 4c, d). Indeed, VIM, a prominent EMT gene [63, 64], was upregulated in MCF10A-TRAM2 and MCF10A-YAP^5SA^ cells compared to control cells based on our proteomics data (Additional file 6: Table S5). This observation was also confirmed by immunoblot analysis (Additional file 1: Fig. S6A).

To investigate the contribution of TRAM2 to YAP-induced EMT phenotype, we suppressed its expression (TRAM2-KO) in MCF10A-YAP^5SA^ cells. By mass spectrometry analysis, we found few differentially expressed genes, of which 21 proteins were downregulated and only 3 were upregulated by both knockdowns (Fig. 4e, f; Additional file 7: Table S6). Interestingly, the strongest shared significant category in both MCF10A-YAP^5SA^-TRAM2-KOs gene set enrichment analyses was reduced EMT signaling (Fig. 4g, h). We confirmed this observation by immunoblot analysis of VIM in MCF10A-YAP^5SA^-TRAM2-KOs and control cells (Additional file 1: Fig. S6B). A similar loss-of EMT signature was also observed in MDA-MB-231-TRAM2-KOs cells, albeit with a smaller magnitude (Additional file 1: Fig. S6C, D; Additional file 8: Table S7). Thus, TRAM2 might play a key function in YAP-mediated EMT.

**Fig. 5:**
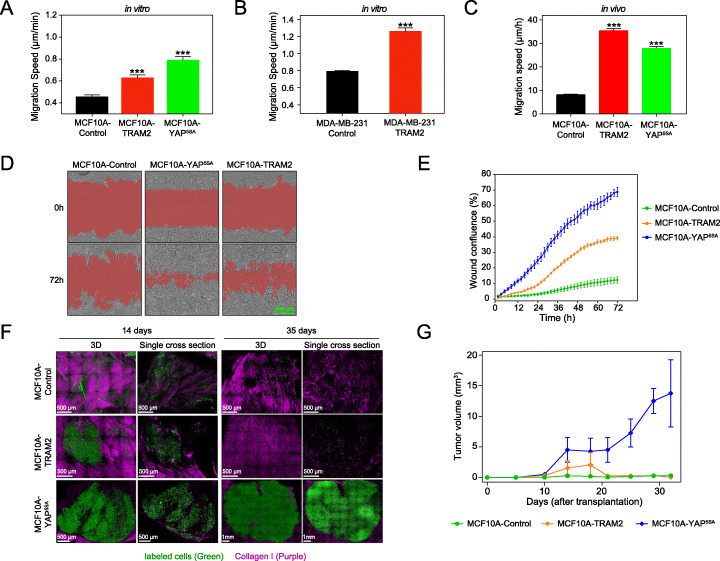
TRAM2 stimulates cell migration and invasion. **a**, **b** In vitro migration assays of MCF10A and MDA-MB-231 cells transduced with the indicated lentiviral vectors. Cells were labeled with GFP for tracking cells under the microscope. Error bars indicate SD from counted cells in each condition (for **a**: Control: *n* = 16962; TRAM2: *n* = 5095; YAP: *n* = 17421; for **b**: Control: *n* = 18448; TRAM2: *n* = 8219). ***P < 0.005, two-tailed Student’s *t* test. **c** In vivo migration assays of GFP-labeled MCF10A cells transduced with the indicated lentiviral vectors. Cells were injected into the fat pad of NOD-*scid* IL2Rgamma^null^ female mice, and 14 days after transplantation cells were tracked by intravital imaging for at least 6 h. In vivo migration speed was tracked for cells that could be followed for a minimum of 4 h. Error bars indicate SEM from 1119 control cells (*N* = 4 tumors), 680 TRAM2 cells (*N* = 4 tumors), and 1746 YAP cells (*N* = 4 tumors) respectively. ***P < 0.005, Welch-corrected two-tailed Student’s *t* test. **d** Representative overview images of Matrigel-coupled wound-healing invasion assays of MCF10A cells transduced with the indicated vectors at 0 and 72 h. **e** Wound confluence was calculated using IncuCyte system based on 4 independent Matrigel-coupled wound-healing invasion assay experiments, as in **d**. **f** Representative overview images of mammary tumors after injection of MCF10A, MCF10A-YAP^5SA^, and MCF10-TRAM2 cells (GFP labeled) in the mammary fat pad of female mice 14 days and 35 days after transplantation. Shown are 3D (left-hand panels) and single cross section images (right-hand panels). Purple, collagen; green, GFP fluorescence from injected labeled cells. **g** MCF10A cells were transduced with the indicated lentiviral vectors, and injected into the mammary fat pad of female NOD-*scid* IL2Rgamma^null^ mice. Tumor growth kinetics were measured using an external caliper. Error bars indicate SD (*N* = 4 tumors per condition)

In light of the association between YAP, TRAM2, and EMT, we examined the migratory and invasiveness capacity of MCF10A-TRAM2 cells. Interestingly, in vitro cellular migration assays indicated that TRAM2 expression induces a similar, albeit with a lower extent, migratory phenotype as MCF10A-YAP^5SA^ cells (Fig. 5a). Immunoblot analysis of TRAM2 confirmed VIM induction in MCF10A-YAP^5SA^ and MCF10A-TRAM2 compared to MCF10A-Control cells (Additional file 1: Fig. S6A). Additionally, even in the context of the highly migratory MDA-MB-231 cells with activated YAP signaling [44, 45], the ectopic expression of TRAM2 boosted cellular migration (Fig. 5b). Also, loss of TRAM2 slightly, but significantly, compromised the migratory capacity of MCF10A-YAP^5SA^ as well as MDA-MB-231 cells (Additional file 1: Fig. S6E, F). To confirm our findings in an in vivo setting, we orthotopically transplanted MCF10A-control, MCF10A-YAP^5SA^, and MCF10A-TRAM2 cells in the fat pad of immunocompromised (NOD-scid Il2ry^null^B2m^null^) female mice. Two weeks later, we performed intravital time-lapse imaging to measure the migratory properties of the transplanted cell populations. Here too, we observed a strong migratory induction by both activated YAP and TRAM2 (Fig. 5c). We further complemented the induced EMT phenotype with invasiveness tests using a Matrigel-coupled wound-healing assay. Figure 5d and e show that MCF10A-TRAM2 cells, like their YAP^5SA^ counterparts, presented a higher invasive characteristic compared with control. The genetic link between TRAM2 and YAP, their co-expression in cancer datasets, and the induction of similar phenotypes (EMT, migration and invasion) prompted us to examine the effect of TRAM2 on in vivo tumor growth. We therefore injected MCF10A-TRAM2, MCF10A-YAP^5SA^, and MCF10A-control cells into the fat pad of immunocompromised mice and monitored tumor-forming capacity. While MCF10A-YAP^5SA^ cells activated a powerful oncogenic growth phenotype and induced tumors in mice, as reported before [29], MCF10A-TRAM2 cells only initiated a transient tumorigenic expansion that regressed 20 days later (Fig. 5f, g). Altogether, our results demonstrate that TRAM2 stimulates cell proliferation, migration, and invasion, but is insufficient to maintain oncogenic growth in vivo. A full-blown tumor growth requires the activation by YAP of other targets than TRAM2.

### FSTL-1 is a key client of TRAM2 in EMT

**Fig. 6:**
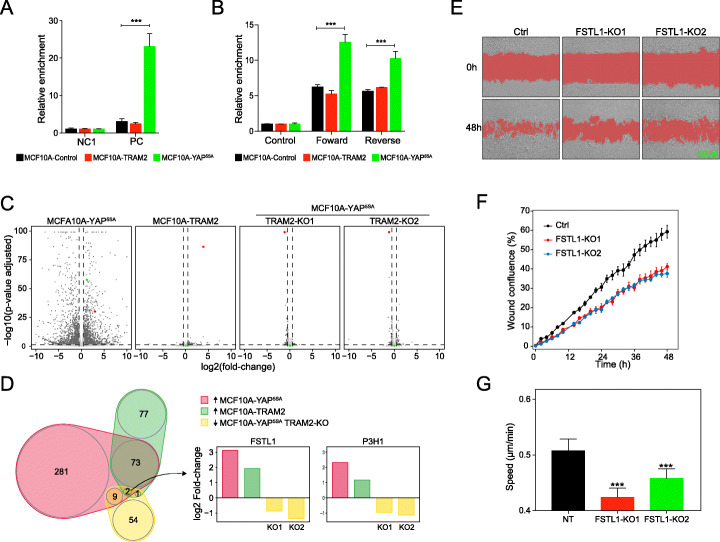
FSTL1 links YAP and TRAM2 to EMT. **a** ChIP-qPCR was used to quantify YAP binding to the indicated regions. NC1 and PC (promoter of ANKRD1) are negative and positive control regions, respectively. Relative YAP-binding enrichment was calculated and normalized to NC1 and input. Error bars indicate SD from three biological replicates. ***P < 0.005, two-tailed Student’s *t* test. **b** The indicated cell populations were subjected to luciferase assays using Enhancer E. Error bars indicate SD from three biological replicates. ***P < 0.005, two-tailed Student’s *t* test. **c** Volcano plots of RNA-Seq analyses in MCF10A cells transduced with the indicated vectors. The data was compared and normalized to control cells (Empty-OE or non-targeting sgRNA). Dark dots are the genes that were significantly upregulated or downregulated in RNA level compered to control with cutoff of absolute LFC > 0.5 and adjusted *p* value < 0.05. YAP is labeled green and TRAM2 red. **d** Venn diagrams representing the intersection of proteins that were upregulated in MCF10A-TRAM2 and MCF10A-YAP^5SA^ cells (raw *p* value < 0.05 and log2 fold-change > 0.7) and were downregulated in MCF10A-YAP^5SA^ cells upon TRAM2-KO1 and TRAM2-KO2 (raw *p* value < 0.05 and log2 fold-change < − 0.7). **e** Representative overview images of Matrigel-coupled wound-healing invasion assays of MCF10A-TRAM2 cells transduced with the indicated vectors at 0 and 48 h. **f** Wound confluence was calculated using IncuCyte system based on 4 independent Matrigel-coupled wound-healing invasion assay experiments, as in **e**. **g** In vitro migration assays of MCF10A-TRAM2 cells transduced with the indicated lentiviral vectors. Cells were labeled with GFP for tracking cells under the microscope. Error bars indicate SD from counted cells in each condition (NT: *n* = 7303; FSTL1 KO1: *n* = 7051; FSTL1 KO2: *n* = 5368). ***P < 0.005, two-tailed Student’s *t* test

We next sought to investigate the underling mechanism by which TRAM2 induces cellular migration and invasion. First, we considered a feedback mechanism in which TRAM2 activates YAP and indirectly induces related phenotypes. To test this option, we performed ChIP-qPCR experiments using anti-YAP antibody in MCF10A-TRAM2, YAP^5SA^, and control cells. While the expected strong and significant increase of YAP-binding in the PC region (positive control region: the promoter of ANKRD1) was observed in MCF10A-YAP^5SA^ cells, MCF10A-TRAM2 cells were as negative as control (Fig. 6a). The lack of YAP activation by TRAM2 was confirmed by luciferase-reporter assays using a YAP-target region (Enhancer E, Fig. 6b). Thus, TRAM2 is likely to act downstream of YAP pathway. To examine this option, we first studied the effect of ectopic expression of TRAM2 and activated YAP (YAP^5SA^) in MCF10A cells and TRAM2 knockout in MCF10A-YAP^5SA^ cells. Corresponding changes in TRAM2 expression were confirmed by immunoblot analysis (Additional file 1: Fig. S6A, B). At the mRNA level, activated YAP induced vast expression changes, as expected from YAP function as a master regulator of transcription (Fig. 6c, Additional file 1: Fig. S7A; Additional file 9: Table S8), which was in accordance with the changes observed at the protein level (Fig. 4a, Additional file 1: Fig. S7A). In contrast, very little mRNA expression changes were detected in MCF10A-TRAM2 cells, as well as by TRAM2-KOs in MCF10A-YAP^5SA^ cells, suggesting that TRAM2 functions at the posttranscriptional level (Fig. 6c, Additional file 1: Fig. S7B, C; Additional file 9: Table S8; Additional file 10: Table S9).

TRAM2 is a component of the translocon, a gated macromolecular channel that couples mRNA translation of nascent secretory and membrane proteins to translocation across the endoplasmic reticulum (ER) membrane [40–42]. Thus, TRAM2 may function as a translational regulator of one or more components of the YAP pathway. We therefore inferred that the key YAP-relevant targets of TRAM2, at the protein level, should be upregulated both in TRAM2 and activated YAP expressing MCF10A cells and downregulated upon TRAM2-KOs in MCF10A-YAP^5SA^ cells. Differential expression analysis between those conditions identified only two proteins that meet the requirements above (FSTL-1 and LEPRE1/P3H1, Fig. 6d). Translocon proteins recognize their clients through a signal peptide (SP) emerging from the N-terminus of newly synthesized proteins [65–67]. Indeed, both FSTL-1 and LEPRE1/P3H1 possess signal peptide, indicating they are likely key direct clients of TRAM2. Interestingly, P3H1 is an enzyme member of the collagen prolyl hydroxylase family. It localizes to the endoplasmic reticulum and its activity is required for proper collagen synthesis and assembly [68]. This is strongly in line with a previous report that TRAM2 is necessary for collagen type I synthesis [69]. Moreover, it has been reported that collagen contributes to cancer progression, such as invasion, metastasis [70–72] which is also in line with the EMT induced by TRAM2. To functionally interrogate FSTL-1 in TRAM2-induced phenotype, we initially validated our proteomics and RNAseq data using qRT-PCR and immunoblots, showing that TRAM2-KOs affect FSTL-1 protein levels without altering its mRNA levels in MCF10A-YAP^5SA^ cells (Fig. 6c, d; Additional file 1: Fig. S7D). To solidify this observation, we then validated that TRAM2-KOs affect FSTL-1 protein level in MDA-MB-231 cells (Additional file 1: Fig. S7E). Next, we knocked FSTL-1 out in MCF10A-TRAM2 cells and examined cellular migration and invasion. These experiments indicated that FSTL-1 is important for maintaining cellular invasion (Fig. 6e, f) and migration (Fig. 6g) phenotypes in MCF10A-TRAM2 cells. Reduced FSTL-1 expression by FSTL-1 KOs was confirmed by western blot (Additional file 1: Fig. S7F). These results are consistent with the observation that FSTL-1 promotes colorectal cancer metastasis via activating the focal adhesion signaling pathway [73].

### High TRAM2 correlates with poor patient survival probability

**Fig. 7:**
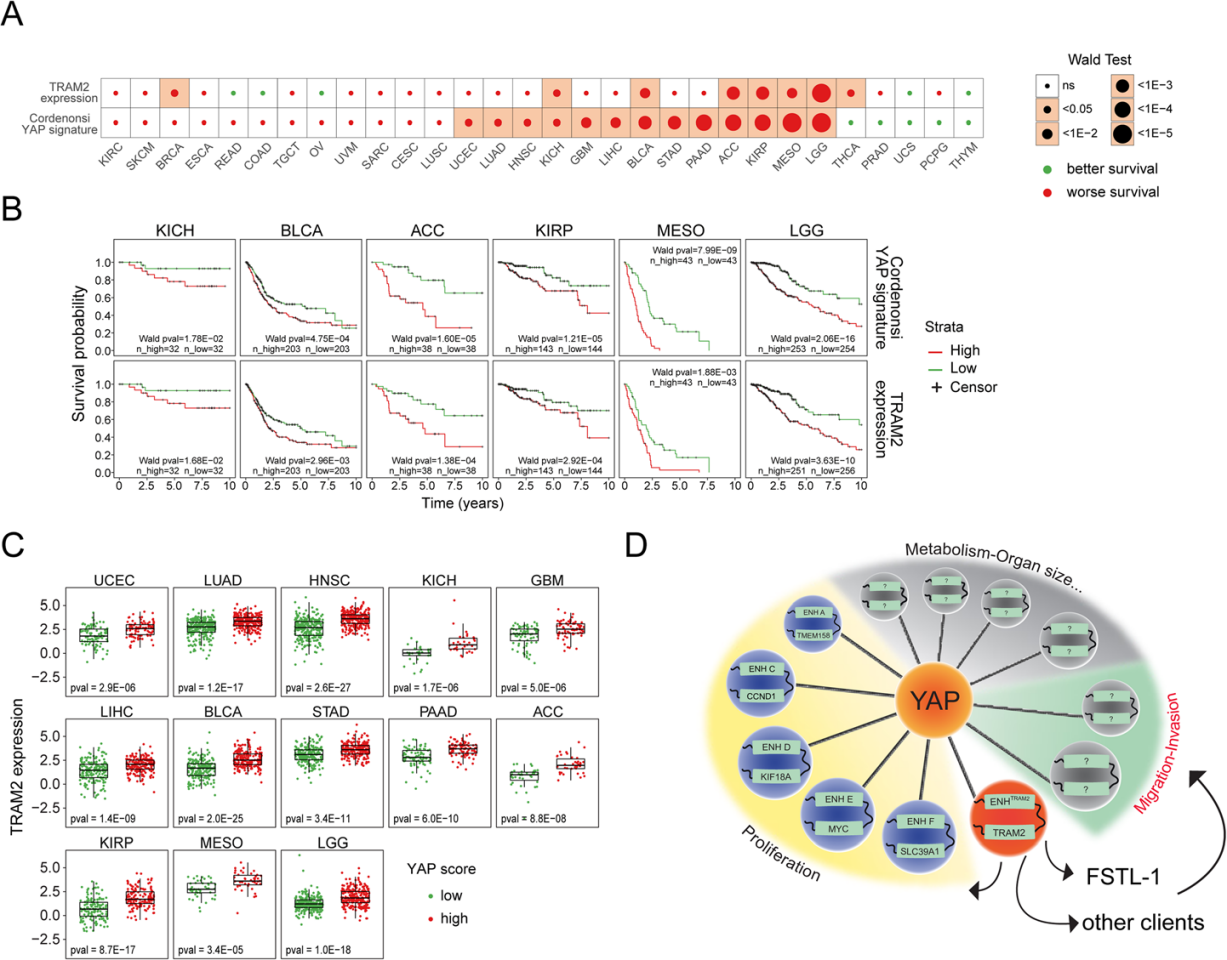
High TRAM2 expression is associated with poor cancer patient survival. **a** The TCGA Pan-Cancer cohort was used to correlate the expression of TRAM2 and YAP gene signature score [47] with survival probability of cancer patients. The heatmap summarizes the results of Cox regression analysis using either TRAM2 expression (log2(TPM)) or YAP gene signature score. **b** Kaplan-Meier plots of TCGA studies with significant survival correlation with both TRAM2 expression and YAP gene signature score. Patients were classified as high (larger or equal than the median; red line) or low (lower than the median; green line) for TRAM2 expression (log2(TPM)) and YAP gene signature score. Wald pval, *p* value of Cox regression calculated in **a**; n_high, number of patients in category “high”; n_low, number of patients in category “low.” Censors were labeled with a black cross. **c** Boxplots showing TRAM2 expression (log2(TPM)) in patients classified as high (larger or equal than the median; red dots) or low (lower than the median; green dots) YAP gene signature score in TCGA studies with significant survival correlation with YAP gene signature score in **a**. **d** A model for YAP-mediated enhancers network uncovered in this manuscript

In light of the key role of TRAM2 in YAP-mediated migration and invasion phenotypes, we investigated the association of its expression with cancer patient survival from the TCGA database. This identified a positive correlation between tumor TRAM2-expression and poor survival probability of patients in 8 TCGA studies. As expected from TRAM2 being a direct target of YAP, 75% of the studies (6 out 8) that show survival correlation with TRAM2 expression also display a similar level of correlation with the YAP gene signature score (Fig. 7a, b). Moreover, in 100% of the TCGA studies where the YAP gene signature score is significantly associated with poor prognosis, TRAM2 is significantly elevated in tumors with high YAP gene signature score compared to low (Fig. 7c). Altogether, these data support a key role of TRAM2 as a mediator of YAP-induced tumor aggressiveness and poor patient survival probability.

## Discussion

Over the past decades, numerous YAP target genes have been discovered and characterized as critical players in YAP-mediated proliferation and oncogenesis. However, a comprehensive view of the transcriptional function provoked by YAP that is required for tumor proliferation and aggressiveness remained largely unknown. The advance in CRISPR-Cas9 technologies enables us to specifically target regulatory DNA elements across the non-coding human genome. The development of functional genetic screening approaches for transcriptional enhancers provides the means to obtain such knowledge. We inferred that targeting enhancers, rather than gene bodies, specifically intervenes with transcription activation, but circumvents the disruption of essential functions of their target genes that can greatly hamper their identification by functional genetic screening of coding genes. Indeed, our screen has identified seven YAP-regulated enhancers and pinpointed their target genes (Figs. 1, 2). Of those, at least three genes (MYC, CCND1, and KIF18A) are essential for cell viability (DepMap, https://depmap.org/portal/) and thus unlikely to be selected in comparable conventional CRISPR functional genetic screens targeting coding sequences. Together with the estimation that the false negative rate discovery of functional YAP regulatory elements in our assay is below 20% (Fig. 1), we conclude that functional genetic screening of regulatory DNA elements is a very robust technology to decipher YAP function.

Compared with activated YAP, the biological effect of each identified enhancer on cell proliferation was relatively small, suggesting that YAP provokes a network of targets to establish a solid phenotype. Our results indicated that the activation of at least six genes is required for YAP-mediated proliferation (Figs. 1, 2, and 3). We focused on TRAM2 in this manuscript because of its unknown link with YAP and cancer progression. From the remaining five, only the roles of MYC [50–52] and CCND1 [53–56] as inducers of cell proliferation and cancer progression in connection with YAP were very well established. The contribution of TMEM158, KIF18A, and SLC39A1 to cell proliferation or cancer progression was established already [57–62], but how exactly they integrate in the YAP signaling pathway is currently unknown. Further investigation is required to explore the exact roles of these genes within the YAP oncogenic signaling network and to establish their roles in cancer development.

Surprisingly, proteomic analysis indicated a stronger link of TRAM2 to YAP and its role in promoting an epithelial to mesenchymal transition (EMT) phenotype [29, 43] (Fig. 4). EMT is associated with cancer resistance and aggressive behavior, and the fact that TRAM2 plays a causal role in these phenotypes (Figs. 4, 5, and 6) indicates that TRAM2 is a key determinant in YAP signaling. Gene expression datasets of many different cancer types confirmed this conclusion by pinpointing a strong link between TRAM2 and YAP gene expression signature, and a strong association with poor cancer patient survival (Fig. 2b and 7a, b).

TRAM2 functions to translocate proteins from the cytosol into the ER. This translocation depends on an N-terminal signal peptide and links subcellular localization to mRNA translation. The correct docking of a client N-terminal signal peptide stimulates protein production in a process termed co-translation. We used this feature to identify TRAM2 clients using mass spectrometry, as loss of TRAM2 should lead to reduced amounts of its direct client proteins but have no impact on its mRNA levels, and identified FSTL-1 as a key functional client of TRAM2 and activated YAP. We demonstrated that the expression of FSTL-1 is required for full-blown induction of cellular migration and invasion induced by TRAM2. It has been reported that FSTL-1 promotes EMT [74]. Moreover, FSTL-1 was shown to promote metastasis and chemo-resistance in esophageal squamous cell carcinoma through NFκB-BMP signaling crosstalk [75] and to promote colorectal cancer metastasis via activating the focal adhesion signaling pathway [73]. Nevertheless, while the effect of FSTL-1 on TRAM2-induced EMT was highly significant, its knockout did not completely abolish migration, clearly indicating that other TRAM2 direct or indirect clients are required for this process too.

Our experiments using intravital imaging indicated that TRAM2 could recapitulate the cell invasiveness behavior of activated YAP in vivo. In contrast, TRAM2 was only sufficient to initiate tumor growth but not to sustain its expansion. This is in line with our genetic screening results indicating that at least seven different YAP regulatory enhancers are required for full oncogenic proliferation. Thus, while our observations tightly tied TRAM2 to YAP signaling network in cancer, and demonstrated TRAM2’s link to cancer patients’ survival rates, they also indicated that other important targets of YAP are required to sustain tumorigenic expansion in vivo. Undoubtedly, the well-described effects of MYC on global transcription and protein production, CCND1 on cell cycle transition, and KIF18A on chromatin segregation contribute greatly to YAP-mediated in vivo tumor expansion. Thus, the experiments described in this manuscript elucidated a YAP-regulated genetic network of enhancers whose targets form a substantial role in stimulating tumor proliferation and aggressiveness (Fig. 7d).

## Conclusions

Our study provides evidence that YAP is localized to enhancer regions to activate specific gene programs for cell proliferation, migration, and invasion during tumorigenesis. We show that YAP controls cell proliferation via at least seven enhancers and their potential target genes (MYC, CCND1, TRAM2, KIF18A, TMEM158, and SLC39A1). We demonstrate TRAM2 functions as a key novel mediator of YAP-induced oncogenic proliferation and cellular invasiveness.

## Methods

### Cell culture

MCF10A and MCF10A-YAP^5SA^ cells were cultured in DMEM/F12 medium (Gibco), supplemented with 1% penicillin/streptomycin (Gibco), 5% horse serum, EGF 10 ng/ml (Sigma), insulin 10 μg/ml (Sigma), and hydrocortisone 0.5 μg/ml (Sigma). MDA-MB-231, MCF-7, and 293 T cells were cultured in DMEM medium (Gibco), supplemented with 1% penicillin/streptomycin (Gibco) and 10% FCS (Hyclone). For mammosphere formation assay, 1000 cells cm^− 2^ were seeded on ultralow-attachment plates (Costar), in DMEM/F12 supplemented with 10 ng/ml EGF, 5 μg/ml insulin, 0.5 μg/ml hydrocortisone, 52 μg/ml bovine pituitary extract, and B27 supplement. Mammospheres were checked after 10 days.

### Analysis of GRO-seq data

GRO-seq was performed on MDA-MB-231 and MCF-7 cells as reported before [38]. Sequencing reads were aligned to the human genome (hg19) using bowtie2 [76], and transcriptional units (TUs) were inferred using HOMER software [77]. Read counts per TU were calculated using HTseq-count [78]. A total of 76,200 TUs covered by at least 20 reads in at least one sample were detected. TU expression levels were then normalized using quantile normalization to allow comparison between samples. Next, we defined bi-directional TUs as pairs of TUs whose start site is separated by no more than 800 bp and are transcribed on opposite strands (TU+ and TU−). As bi-directional transcription is a hallmark of transcriptional regulatory elements, we refer to these loci as regulatory enhancer elements.

### CRISPR library construction and analysis

We designed a CRISPR library to target the TEAD4 motifs in the potential enhancer regions. For 2439 of the 2902 regions containing TEAD4 motifs, we found an NGG PAM in a location that is expected to induce a Cas9 DNA cleavage within a distance of 3 bp with respect to the motif (that is, the cut is expected to occur within the motif or up to 3 bp from its edges). Overall, we could target 3220 motifs and designed 4871 distinct sgRNAs, including positive and negative controls. To generate a CRISPR-Cas9 lentiviral library (hereafter referred as CRISPR-YAP-enh-lib), the sgRNAs were cloned into pLentiCRISPRv2 using Gibson Assembly from an oligonucleotide pool synthesized by CustomArray Inc. MCF10A and MCF10A-YAP^5SA^ cells were transduced with three independent lentiviral pools of CRISPR-YAP-enh-lib. After selection with puromycin (1 mg/L), half of the transduced cells were harvested at day 0, while the remaining cells were cultured with continuous passaging for 20 days. After 20 days, cells were harvested, genomic DNA was isolated and the integrated sgRNAs were PCR-amplified and submitted for next-generation sequencing (NGS) to quantify their relative abundance. Sequencing was done using single reads of 65 bp on the Hi-Seq2500 platform (Illumina). Sequencing reads were first trimmed to remove adaptor sequences and then aligned to the sgRNA library sequences, using cutadapt and bowtie2 software, respectively[76, 79]. Read counts per sgRNA were then calculated using R language. To assess the relative change between the starting and final cell population, MAGeCK software was employed using the non-targeting negative control sgRNAs for normalization [80]. For a comparison between the two cellular models, the average log2 fold-change obtained from the three replicates was used.

### Luciferase reporter assay

The enhancer E region was PCR amplified from DNA of MCF10A cells and cloned into downstream of Poly-A of the firefly luciferase reporter gene in the pGL3-promoter (Promega) vector. For transfection of these plasmids, 1 × 10^5^ of MCF10A-YAP^5SA^, MCF10A-Control, MCF10A-TRAM2 cells were seeded in 6-well plates. The next day, 200 ng of each construct (pGL3-promoter, pGL3-promoter-enhancer/forward, and pGL3-promoter-enhancer/reverse) were co-transfected with 20 ng of Renilla luciferase reporter construct using Fugene-6 (Promega) following the manufacturer’s protocol. Luciferase activity was measured 24 h post-transfection using the dual-luciferase reporter assay kit (Promega). Cells were lysed directly on the plate with passive lysis buffer for 15 min at room temperature. Firefly and Renilla luciferase activity was measured using a Centro XS3 LB960 machine (Berthold technologies).

### Lentivirus production and infection

293T cells were seeded at the density of 6 × 10^6^ cells per 10 cm dish 1 day prior to transfection. Transfection was performed using PEI (Polyethylenimine, Polysciences), and the medium was refreshed 16 h later. Virus-containing supernatant was collected 48 h post-transfection. Next, virus was filtered through a 0.45 μm membrane (Millipore Steriflip HV/PVDF), snap-frozen and stored at − 80 °C. MCF10A and MCF10A-YAP^5SA^ were infected and selected with the proper antibiotics 48 h after transduction for at least 4 days until no surviving cells remained in an uninfected control plate.

### Mutational load analysis generated by sgRNAs

Genomic DNA of MCF10A and MCF10A-YAP^5SA^ cells transduced with sgRNAs was isolated and the concentration was measured. Five hundred nanograms of the genomic DNA was used for PCR-amplification of the enhancer region. We performed a two-step PCR by introducing the P5 adapter sequence in the first PCR and P7 adapters with the indexes in the second PCR. After the second PCR, the libraries were purified with CleanNA beads (GC Biotech) and quantified on the 2100 Bioanalyzer using a 7500 chip (Agilent). Equimolar amounts of each sample were taken for samples ran on the same lane. Deep sequencing was performed with single reads of 150 bp on the Mi-Seq system with Mi-Seq reagent v2 Nano kit. Sequenced reads were aligned to the amplified enhancer region using Bowtie. Bam files were analyzed to count the number of mutations (mismatches, insertions, or deletions) identified at each location in the enhancer regions.

### RNA-seq library construction and analysis

Total RNA was isolated using Trisure reagent (Bioline) following the manufacturer’s protocol. Briefly, cells were lysed in Trisure, precipitated with isopropanol, and dissolved in RNase-free water. To generate strand-specific libraries, we used the TruSeq Stranded mRNA sample preparation kit (Illumina) following the manufacturer’s instructions. The sequencing libraries were analyzed on a 2100 Bioanalyzer using a 7500 chip (Agilent) and pooled equimolar into a 10-nM multiplex sequencing pool. Enhancer target gene identification (Fig. 2a), RNA-Seq sequenced reads were aligned to the human genome (hg19) using TopHat2 [81]. The number of reads mapped to each annotated gene was counted using HTseq-count [78] and then converted to RPKMs (using GENCODE v25 annotations). RPKM levels were further normalized using quantile normalization, and expression levels in each sample relative to the control non-targeting sample were calculated (in log2 base). The rest of RNA-Seq analysis was done by first removing adapter sequences using cutadapt and then performing gene quantification using Salmon software [82]. Differential expression analysis was performed using R package DESeq2 [83]. Volcano plots and Venn diagrams were made using R language and packages ggplot2 [84] and nVennR [85].

### Genome graphs

For YAP and TEAD4 binding tracks, ChiP-Seq raw reads data from MDA-MB-231 cell line were downloaded from the Sequence Read Archive (accession SRP055170). Raw reads from replicates were merged and subsequently adaptor-trimmed and aligned to the human genome (hg19) using cutadapt and Bowtie2 software [76, 79]. Transcription factor occupancy was then calculated as fold enrichment relative to control samples (IgG ChipSeq) using Macs2 software [86]. For GRO-Seq tracks, aligned reads from GRO-Seq experiments (described above) in MCF-7 and MDA-MB-231 were used to calculate the genome coverage (normalized for sequencing depth) using BEDtools [87]. Layered H3K27ac tracks were downloaded from UCSC browser using the R package Gviz [88]. For gene tracks, the latest Ensembl gene annotation for genome assembly hg19 was used. For TAD tracks, binned (5Kb) chromatin interactions were retrieved from HiC experiments in HMEC cells [89] (https://hicfiles.s3.amazonaws.com/hiseq/hmec/in-situ/combined.hic) using Juicer Tools (with option “Vanilla Coverage” normalization) [90]. For CTCF and RNA Pol-II chromatin interaction tracks, BED formatted interactions from ChIA-PET experiments in MCF-7 cells were downloaded from ENCODE (accessions ENCSR000CAD and ENCSR000CAA) and replicates were combined using a R language and package Genomic Ranges [91]. Genome graphs in Fig. 1e and Fig. S1C, S2B were generated in R language using the Gviz package. Genome graphs in Fig. 2a and Fig. S4A were generated using custom R language with package ggplot2. Gene expression tracks in Fig. 2a and S4A were generated using log2-fold-change gene expression from RNA-Seq experiments in enhancer-knockout cell lines compared to non-targeting infected cells (described above).

### TCGA analysis

For gene expression correlation between enhancer target genes and YAP gene expression signature, gene expression data for the TCGA Pan-Cancer cohort recomputed by the Toil RSEM pipeline were downloaded as transcripts per million (TPM) from the XENA browser (https://toil.xenahubs.net/download/tcga_RSEM_gene_tpm.gz). The downloaded log2 (TPM + 0.001) expression data was used to calculate the Pearson correlation coefficient between each enhancer target gene or nearby gene and a YAP gene expression signature across all TCGA available studies. YAP gene signature score was calculated for every sample using the expression of 57 genes defined in publication [47] employing the ssGSEA method from the R package GSVA [92]. For the YAP gene signature score calculation, genes with log2 (TPM) lower than − 1 were filter out sample wise. Only primary tumor samples were considered and combinations of TCGA studies where the average log2 (TPM) of the gene of interest was lower than − 1 were labeled as “low expression” and discarded for correlation calculation. Pearson correlation coefficient and correlation test (Bonferroni corrected) were computed using R language.

Survival correlation analyses for TRAM2 expression and YAP activity were done employing TRAM2 log2 (TPM) and the previously calculated YAP gene signature scores using the patient overall survival (OS) time computed in [93] and retrieved from XENA browser (https://pancanatlas.xenahubs.net/download/Survival_SupplementalTable_S1_20171025_xena_sp.gz). For every TCGA cancer study, overall survival distribution differences were assessed using Cox regression with R package survival [94]. For selected TCGA studies, survival probability was plot using the Kaplan-Meier estimator between samples with low (lower than the median) and high (higher or equal to the median) TRAM2 expression or YAP gene signature score, using R language.

### Chromatin immunoprecipitation (ChIP) and qPCR

Chromatin immunoprecipitations were performed as previously described [95–97]. Cells were fixed with formaldehyde (1%) for 10 min and subsequently quenched with glycine. Following that, samples were lysed in 1.5 ml Bioruptor Pico Microtubes and sonicated for at least 13 cycles of 30 s on, 30 s off using Diagenode Bioruptor Pico. We used antibodies against YAP (EP1674Y, ab52771, Abcam). For YAP ChIPs, 5 μg of antibody was conjugated with 50 μL Protein A magnetic beads. The immunoprecipitated DNA and input DNA were processed for qPCR to measure the YAP binding to specific regions by using specific primers. Primers are listed in Table S10.

### Chromatin conformation capture (3C) analysis

10 × 10^6^ cells were harvested in PBS for each 3C sample. Cells were centrifuged at 300×*g* for 5 min at RT and resuspended PBS/10% FBS. Then, cells were incubated with equal volume of 4% formaldehyde (2% end concentration) for 10 min and quenched with 2 M glycine solution (0.2 M end concentration), followed by centrifugation at 300×*g* for 5 min at 4 °C. Cell pellet was then resuspended in PBS/10% PBS and centrifuged at 300×*g* for 5 min at 4 °C. The supernatant was then discarded and snap-frozen, stored at − 80 °C. The cell pellet was lysed in 3 mL lysis buffer (50 mM Tris-HCl pH 7.5, 0.5% NP-40, 1% Triton X-100, 150 mM NaCl, 5 mM EDTA, protease inhibitor cocktail (Roche)) for 1.5 h at 4 °C, followed by centrifugation at 1000×*g* for 3 min. The pellet was washed once in 1.2× restriction buffer and resuspended again in 500 μL of 1.2× restriction buffer. 15 μL of 10% SDS was added to the suspension and incubated at 37 °C while shaking at 400 rpm. 75 μL of 20% Triton X-100 was added to the suspension and incubated at 37 °C while shaking at 400 rpm. The samples were then centrifuged at 1000×*g* for 3 min and resuspended in 500 μL of 1× restriction buffer. The digestion was performed with addition of 200 U of MboI (NEB) at 37 °C overnight. The digestion efficiency was assessed the next day on agarose gel. The enzyme was then inactivated at 65 °C for 20 min and then samples were centrifuged at 1000×*g* for 3 min to remove the restriction buffer. The pellet was resuspended in 7 mL of 1× ligation buffer, and the ligation was performed with addition of 50 U of T4 DNA ligase (5 U/μl) (Thermo Scientific) at 16 °C overnight. Again, the ligation efficiency was examined on agarose gel. De-crosslinking was performed by addition of 30 μL of protease K (Roche) at 65 °C overnight. To remove residual RNA, 15 μL of RNaseA cocktail (Ambion) was added to the samples and incubated at 37 °C for 45 min. DNA was recovered by adding 7 mL of isopropanol and 70 μL of NucleoMag® P-Beads (Bioke) and incubated for 30 min at room temperature. The samples were centrifuged for 3 min at 1000×*g* and washed with 80% ethanol twice. Finally, the beads were dried and eluted in 300 μL of 10 mM Tris-HCl pH 7.5. To assess the physical interactions between Enhancer^TRAM2^ and target regions, we designed a constant primer (E) that amplifies the Enhancer^TRAM2^ region overlapping the junction created by MboI enzyme. For each assessed region, we designed two primers (reverse and forward) to examine the interactions with Enhancer^TRAM2^. Finally, PCR products were resolved on 2% agarose gel. To examine the sequences of the PCR products, DNA bands were cut, isolated, and sanger-sequenced.

### Plasmid cloning

YAP^5SA^ and YAP^5SA-S94A^ lentiviral vectors were sub-cloned from pCMV-FLAG-YAP-5SA and pCMV-FLAG-YAP-5SA-S94A (obtained from Addgene#27371 and #33103) into pLenti-hPGK-BlastR (CpG-low)-hEF1(CpG-free) backbone plasmid. TRAM2 open reading frame was PCR amplified from RNA and cloned into pCDH-CMV-MCS-EF1-Puro vector (obtained from System Bioscience; CD510B-1). To clone pCDH-H2B-GFP vector, H2B-GFP was cloned into a pCDH-CMV vector (Agami lab). TRAM2-KOs and FSTL1-KOs plasmids were cloned by following the protocol from addgene (GeCKO, zhangLab). The primers are listed in Table S10.

### H2B-GFP cell generation and competitive proliferation assay

Cells were infected with pCDH-H2B-GFP virus at a multiplicity of infection (MOI) of ~ 0.3. Then, GFP positive cells were sorted by FACS. MCF10A, MCF10A-YAP^5SA^, and MDA-MB-231 cells were infected with indicated sgRNAs. Separately, we generated polyclonal MCF10A, MCF10A-YAP^5SA^, and MDA-MB-231 cells stably expressing H2B-GFP. GFP expressing cells were mixed in a 1:3 ratio with cells containing individual sgRNAs. The percentage of GFP-expressing cells was assessed by flow cytometry at the beginning of the experiment (day 0) and at day 6 and day 12. For every condition, 10,000 events were recorded, and the data were analyzed using FlowJo software.

### Proteomics

For proteome analysis, cells pellets were lysed in boiling guanidine lysis buffer as described by Jersie-Christensen et al [98]. Protein concentrations were determined with a Coomassie (Bradford) Protein Assay Kit (Pierce) according to the manufacturer’s instructions. Proteins were digested with Lys-C (Wako, enzyme/protein ratio 1:100) for 2 h at 37 °C, after which samples were diluted to 2 M GuHCl and digested overnight with trypsin (Sigma-Aldrich, enzyme to protein = 1:50). Digestion was quenched by the addition of trifluoroacetic acid (TFA, 1% final concentration) and peptide samples were desalted on Sep-Pak C18 cartridges (Waters, Massachusetts, USA). After elution, aliquots were collected for proteome analysis. All samples were dried using a SpeedVac and stored at − 80 °C until LC-MS/MS analysis.

### Mass spectrometry and proteome data analysis

Prior to mass spectrometry analysis, peptides were reconstituted in 2% formic acid. Peptide mixtures were analyzed by nanoLC-MS/MS on an Orbitrap Fusion Tribrid or Q Exactive HF-X Hybrid Quadrupole-Orbitrap Mass Spectrometer equipped with an EASY-nLC 1200 system (Thermo Scientific). Samples were directly loaded onto the analytical column (ReproSil-Pur 120 C18-AQ, 1.9 μm, 75 μm × 500 mm, packed in-house). Solvent A was 0.1% formic acid/water, and solvent B was 0.1% formic acid/80% acetonitrile. Samples were eluted from the analytical column at a constant flow of 250 nl/min. For single-shot proteome analysis, a 3h gradient (Q Exactive) or 4h gradient (Orbitrap Fusion) was employed containing a gradual, non-linear increase from 7% B to 60% B and finishing with a 15-min wash. Proteome data (RAW files) were analyzed by Proteome Discoverer (version 2.3.0.523, Thermo Scientific) using Percolator and standard settings. MS/MS data were searched against the human Swissprot database (20,417 entries, release 2019_02) with Sequest HT. The maximum allowed precursor mass tolerance was 50 ppm and 0.06 Da for fragment ion masses in the case of Q Exactive data and 50 ppm/0.6 Da for Orbitrap Fusion data. False discovery rates for peptide and protein identification were set to 1%. Trypsin was chosen as cleavage specificity allowing two missed cleavages. Carbamidomethylation (C) was set as a fixed modification, while oxidation (M), acetyl (Protein N-term), and deamidation (NQ) were used as variable modifications. The Proteome Discoverer output file containing LFQ abundances and PSM filtered for Xcorr> 1 was loaded into Perseus (version 1.6.5.0) [99]. Abundances were Log2-transformed, and proteins were filtered for at least two out of three valid values in both conditions for each comparison. Differential expression analysis was performed using linear model fitting and empirical Bayes moderated *t*-statistics from limma R package combined with Benjamini-Hochberg multiple test correction [100]. Gene Set Enrichment Analysis was performed on the proteomics data using pre-ranked GSEA with the metric − log10(*p* value)*sign (LFQ differences) using GSEA software [101]. Bubble plots and volcano plots were generated using R language with package ggplot2. Venn diagrams to compare the overlap between MCF10A-YAP^5SA^ and MCF10A-TRAM2 were generated using the R package nVennR [85]. Significantly changed proteins which overlap between MCF10A-YAP^5SA^ and MCF10A-TRAM2 were calculated using Fisher’s exact test from the GeneOverlap R package.

### In vitro migration assay

2.5 × 10^5^ cells were seeded on fibronectin (0.1 mg/mL)-coated glass bottom dishes (35 mm^2^) in culture medium without phenol red. Experiments were performed 16–24 h after plating. Time-lapse movies were recorded. Cells were labeled with GFP and were imaged using 460 nm excitation (10 mW/cm^2^) for an hour at 1 frame/min.

Migration data were analyzed via a Matlab-based modified Gaussian tracking mixture model, revised from Amat. F. et al [102]. In brief, the time-lapse images were first processed for single-cell (nuclei) detection followed by cell segmentation and tracking, via which coordinates of individual cells over time were recorded and extracted for migration speed analysis. Average speed was calculated from the accumulated migration distance divided by the total time.

### In vitro invasion assay

The 96-well ImageLock™ plate was coated with 50 μL Matrigel (100 μg/mL) after pre-cold on ice and incubate the plate in 37 °C for 2 h followed by aspirating the unsolidified Matrigel. Then, 4 × 10^4^ cells were seeded per well. Next day, the homogeneous 700–800 μm-wide scratch wounds were made in each well on cell monolayers using a WoundMaker™ (Essen Bioscience). Matrigel was diluted to 5 mg/mL with cold PBS and kept on ice to avoid solidification before use. Debris and detached cells were removed by washing with fresh medium twice and the 96-well plate was kept on ice until the plate is cold (4 °C) followed by removing the medium and adding 50 μL prepared cold Matrigel into each well to cover the wound. Put the plate into incubator to solidify the Matrigel which provides a 3D tissue-like environment for cell invasion assay. Add 100 μL fresh medium to each well and put the plate into IncuCyte. Real-time images were taken every 2 h for 48 h or 72 h in total. Wound closure was quantified using the relative wound density metric by the instrument software.

### Western blots

Cells were harvested by scraper and lysed with RIPA buffer supplemented with 1× complete protease inhibitor cocktail (Roche) following the manufacturer’s protocol. Protein concentrations were determined using a Pierce BCA protein assay kit (Thermo Scientific). Lysates were separated on SDS-PAGE gels and transferred. Membranes were immunoblotted with the following antibodies: YAP (ab52771, EP1674Y, abcam; 1:1000), TRAM2 (ab109176, EPR2658, abcam; 1:5000), FSTL-1 (ab11805, abcam, 1:1000), Vimentin (550,513, BD Biosciences, 1:2000), HSP90 (610,418, BD Biosciences, 1:5000), and GAPDH (sc-32,233, Santa Cruz, 1:2000). Protein bands were visualized using corresponding secondary antibodies (Dako) and ECL reagent (GE Healthcare).

### Mouse experiments

NOD-*scid* Il2Rgamma^null^-female mice were obtained from Jackson Laboratories. All animal experiments were approved by the Animal Welfare Committee of the Netherlands Cancer Institute (NKI) in accordance with national guidelines. Animals were maintained in the animal department of NKI, housed in individually ventilated cages (IVC) under specific pathogen-free (SPF) conditions, and received food and water ad libitum. Mice were used for experiments between 8 and 25 weeks of age. MCF10A Control-, MCF10A TRAM2-, and MCF10A YAP- cells labeled expressing H2B-GFP were transplanted in the fat pad of the 4th mammary gland of 8–12-week old NOD-*scid* Il2Rgamma^null^ female mice. For each cell line, 1 × 10^6^ cells diluted in 50 μL sterile PBS and 50 μL Cultrex PathClear Reduced Growth Factor (RGF) BME, Type 2 (Amsbio) were injected under aseptic conditions, while animals were sedated with a 2% isoflurane/compressed air mixture. Tumor size was monitored twice a week, and tumors were used for imaging and further processing 14 or 35 days after injection.

### Intravital time-lapse imaging

Tumor-bearing mice were sedated using isoflurane inhalation anesthesia (1.5% isoflurane/compressed air mixture). Tumors were surgically exposed through a skin flap, and the mouse was placed in a custom-made imaging box. Isoflurane was introduced through a facemask in the imaging box and ventilated by an outlet on the other side of the box. Imaging was performed on an inverted Leica SP8 Dive system (Leica, Mannheim, Germany) with a MaiTai eHP DeepSee laser (Spectra-Physics) and an InSight X3 laser (Spectra –Physics), using the Leica Application Suite X (LAS X) software. Second harmonic generation (Collagen I) and GFP were simultaneously excited using 860 nm (Mai Tai) and 925 nm (Insight X3) and detected at 400–460 nm and 490–550 nm respectively. First, three-dimensional tile scans of the full visible tumor area were acquired with 2 μm Z-step size. To follow in vivo tumor cell migration, regions of interest in these tumors were selected from the overview scan and were subsequently imaged as three-dimensional volumes every 30 min with a Z-step size of 1 μm over a minimal period of 6 h. All images were acquired with a 25x water immersion objective (HC FLUOTAR L 25x/0.95 W VISIR 0.17, working distance 2.40 mm).

### Mice experiment image analysis

Three-dimensional overview tile scans of the tumors were stitched and processed in the true 3D real-time Rendering LAS X 3D Visualization module (Leica microsystems, Mannheim, Germany). The time-lapse three-dimensional volumes were corrected for XYZ-shift using the Huygens Object Stabilizer module (Scientific Volume Imaging). Individual cells were tracked using the MTrack2 plugin in ImageJ (Stuurman, N., Schindelin, J., Elliot, E., and Hiner, M., http://imagej.net/MTrack2). Only cells that could be followed over a minimal period of 4 h were included in the analysis.

## Supplementary Information

**Additional file 1:** Figures S1-S7 with figure legends.

**Additional file 2: Table S1.** CRISPR-YAP-enh-lib sequences.

**Additional file 3: Table S2.** CRISPR screen analysis.

**Additional file 4: Table S3.** CRISPR screen selected hits.

**Additional file 5: Table S4.** RNA-seq to identify the target genes of validated enhancers.

**Additional file 6: Table S5.** Proteomics data analysis of MCF10A-YAP^5SA^ and MCF10A-TRAM2 cells.

**Additional file 7: Table S6.** Proteomics data analysis of TRAM2-KO in MCF10A-YAP^5SA^ cells.

**Additional file 8: Table S7.** Proteomics data analysis TRAM2-KO in MDA-MB-231 cells.

**Additional file 9: Table S8.** RNA-seq data analysis of MCF10A-YAP^5SA^ and MCF10A-TRAM2 cells.

**Additional file 10: Table S9.** RNA-seq data analysis of TRAM2-KO in MCF10A-YAP^5SA^ cells.

**Additional file 11: Table S10.** Primers for ChIP-qPCR, 3C, q-PCR and gene knockout.

**Additional file 12:** Complete western blot images of all figures in the manuscript.

**Additional file 13:** Review history.

## Data Availability

The raw RNA- and GRO-sequencing data reported in this paper are available at GEO database with accession numbers: GSE139004, GSE137981, GSE147556, GSE147669 [103]. The public ChIP-seq data are available at GEO database with accession number: GSE66081 [29]. Proteomics data are available via ProteomeXchange with identifier PXD018559.
