# Supplementary material for: A comprehensive enhancer screen identifies TRAM2 as a key and novel mediator of YAP oncogenesis
Source: Genome Biol. 2021 Jan 29;22:54. doi: 10.1186/s13059-021-02272-8 (PMC7845134; doi:10.1186/s13059-021-02272-8)
Supplement: Supplementary file 1 — Additional file 1: Figures S1-S7 with figure legends. [file 13059_2021_2272_MOESM1_ESM.pdf]

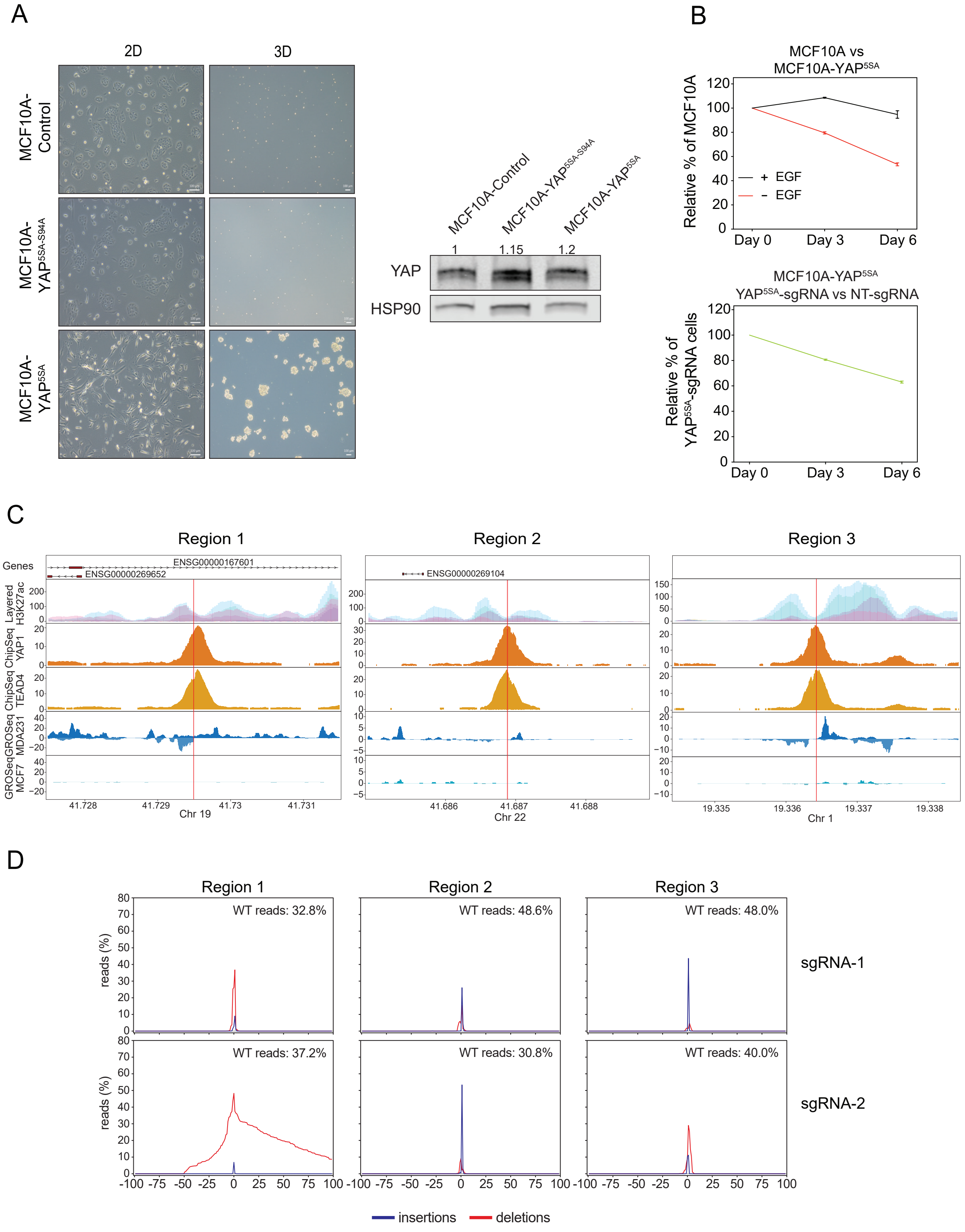

Fig. S1. Generation of the MCF10A-YAP^{5SA} cell system and evaluation of CRISPR efficiency.
(A) Representative images of MCF10A cells transduced with indicated lentiviral vectors in 2D and 3D (mammosphere assay) cultures. Western blot analysis showing YAP and HSP90 (loading control) protein levels. **(B)** Cell proliferation measured by cell competition assays. Upper panel shows cell proliferation of MCF10A-YAP^{5SA} cells compared to MCF10A-control cells in the presence or absence of EGF. Lower plot represents the cell proliferation of MCF10A-YAP^{5SA} infected with a YAP^{5SA}-targeting sgRNA compared to non-targeting sgRNA. **(C)** Genome graphs of the selected regions (1-3, as detailed in Fig. 1B) supplemented with genomic information of the targeting sgRNA sites (red line), H3K27ac levels (an active enhancer marker), YAP and TEAD4 binding, and GRO-seq data obtained from MDA-MB-231 and MCF7 cells. **(D)** PCR-seq analysis of the targeted genomic loci in MCF10A-YAP^{5SA} cells transduced with the indicated sgRNAs. The proportion of wild-type or mutated (deletions or insertions) sequencing reads is indicated as a function of their distance from the sgRNA-induced Cas9 cleavage site.

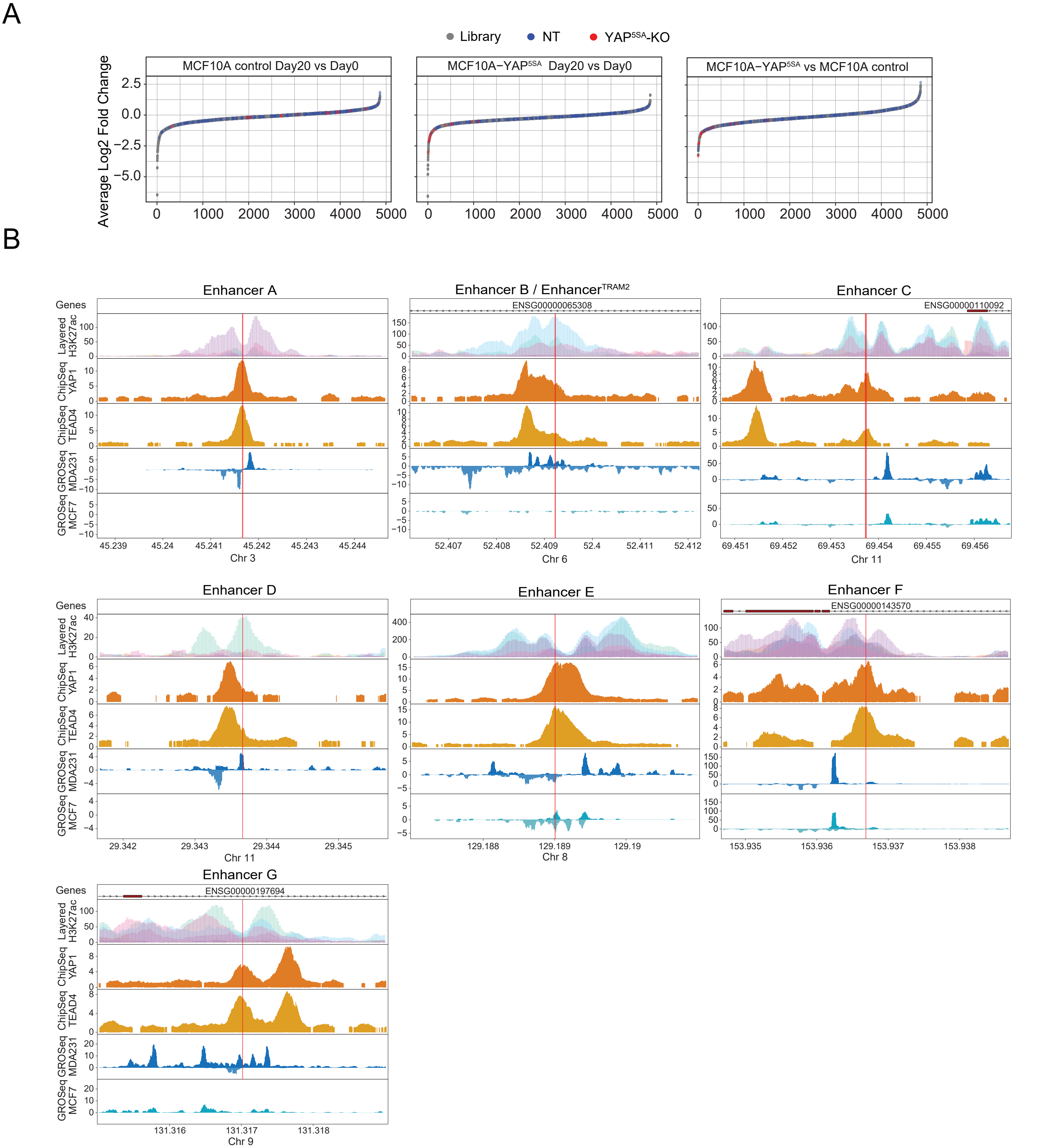

Fig. S2. CRISPR screen analysis and validation of the selected hits.
(A) sgRNAs were sorted by the enrichment score based on the ratio between their abundance at day 20 and day 0 in MCF10A-YAP^{5SA} and MCF10A control cells. The Y-axis indicates the average of log2 fold changes (LFC) of the sgRNA enrichment scores (calculated from 3 biological replicates). Red dots are sgRNAs targeting YAP^{5SA} (positive controls), blue dots are non-targeting sgRNAs (NT), and gray dots are sgRNAs targeting TEAD4 motifs in the genome. **(B)** Genome graphs of selected candidate enhancers supplemented with genomic information of the targeting sgRNA sites (red line), H3K27ac levels (an active enhancer marker), YAP and TEAD4 binding, and GRO-seq data obtained from MDA-MB-231 and MCF7 cells.

A

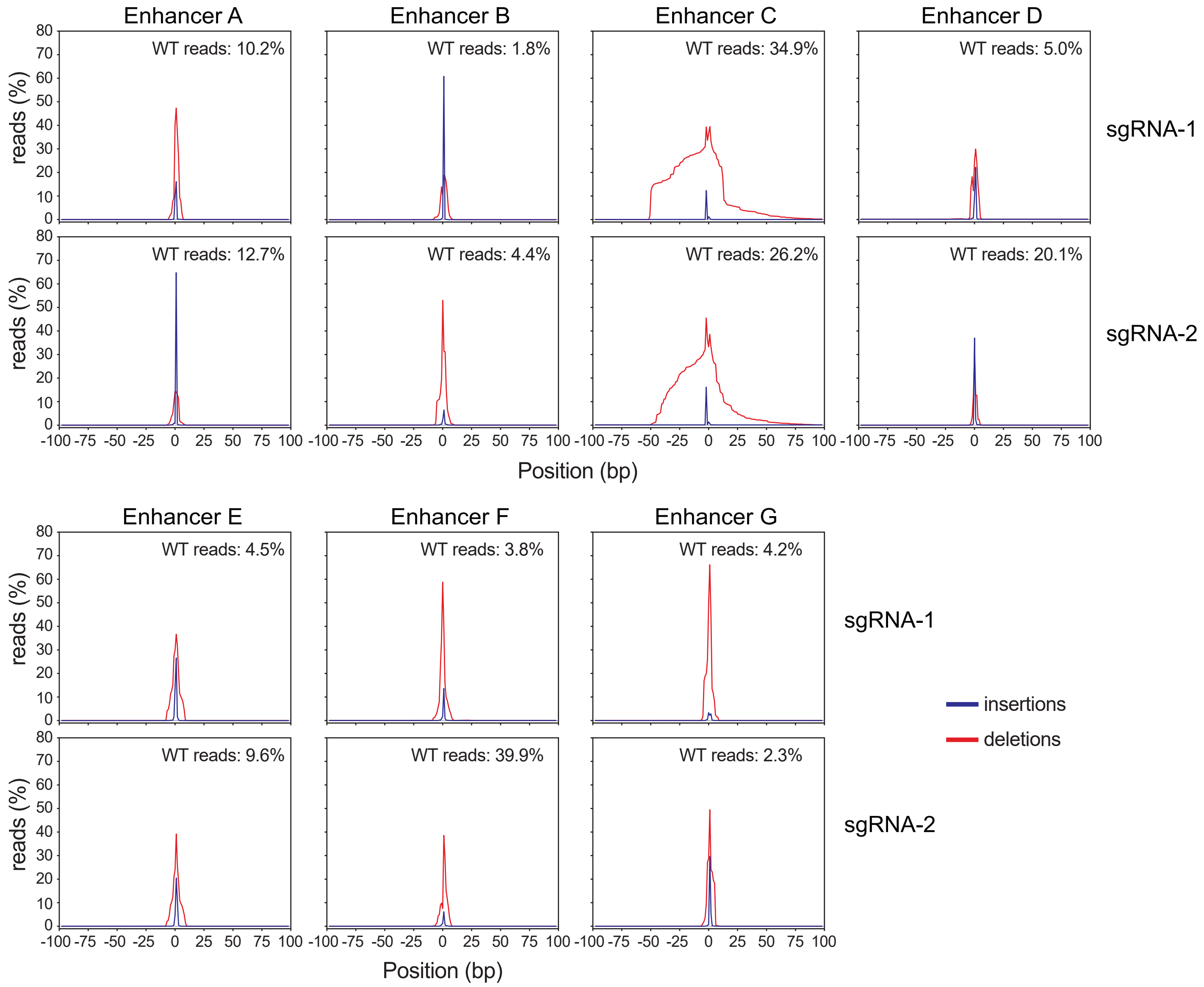

B

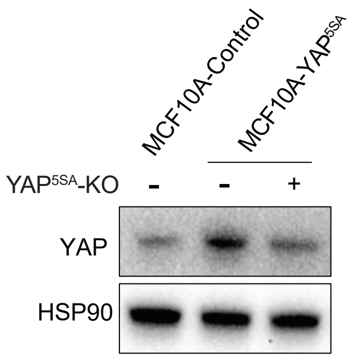

Fig. S3. Validation of the selected hits.
(A) Indel analysis, as detailed in Fig. S1D, of the indicated targeted genomic loci. **(B)** Western blot analysis showing YAP protein levels in MCF10A-YAP^{5SA} cells and its effective knockdown by the YAP^{5SA}-targeting sgRNA. HSP90 was used as loading control.

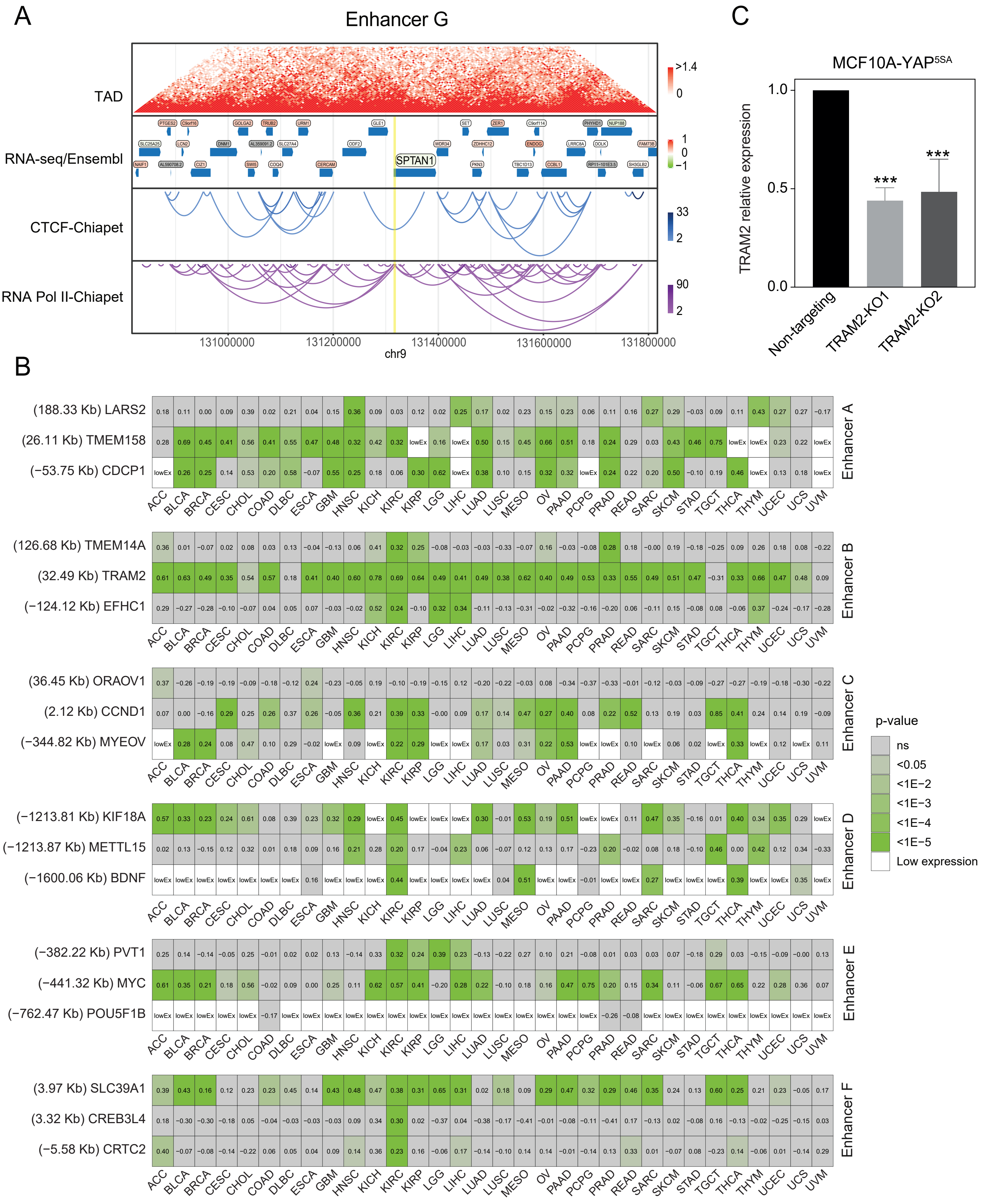

Fig. S4. Target gene identification of YAP-enhancers.

(A) A genomic graph, as detailed in Fig. 2A, centered at the enhancer region G. **(B)** Heatmap plots, as detailed in Fig. 2B, including the Pearson correlation coefficient between nearby genes of each enhancer and the YAP gene signature score in publicly available TCGA RNA-Seq samples from human tumors. Numbers in brackets next to each symbol represent the distance in kb from each gene's promoter to the enhancer region. **(C)** qRT-PCR to measure the TRAM2 expression upon Enhancer^{TRAM2}-KOs in MCF10A-YAP^{5SA} cells. Error bars indicate SD from three biological replicates. ***P<0.005, two-tailed Student's t-test.

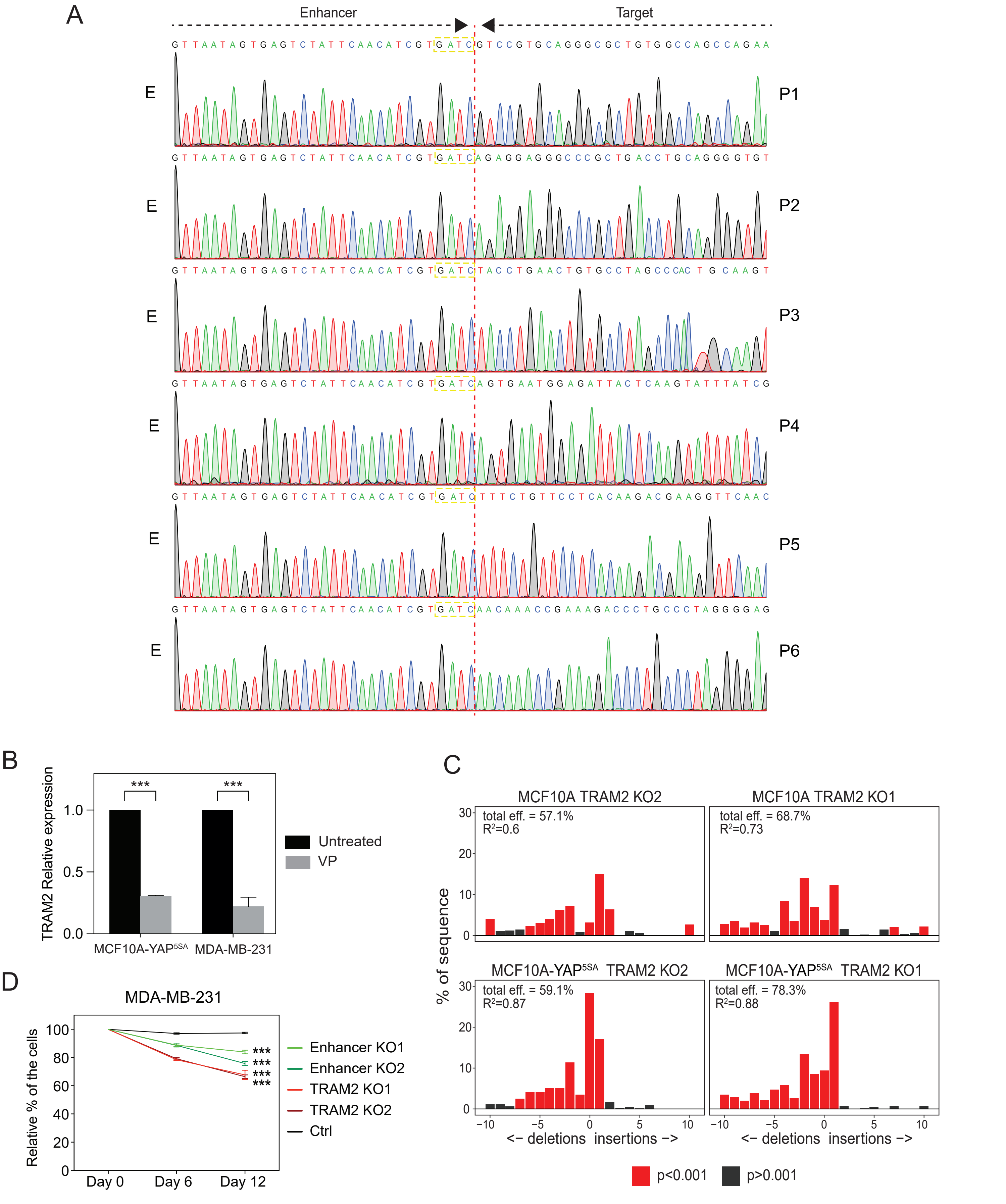

Fig. S5. Confirmation of the 3C PCR results and the effect of knocking out TRAM2 and Enhancer^{TRAM2} in MDA-MB-231 cells. **(A)** DNA fragments isolated from the gel presented in Fig. 3D were sequenced to validate the correct enhancer-target fragments ligation. The MboI restriction site is highlighted in yellow. The PCRs were performed using the indicated primer pairs. **(B)** qRT-PCR to measure the TRAM2 expression upon verteporfin treatment in MCF10A-YAP^{5SA} (2μM) and MDA-MB-231 (10μM) cells. Error bars indicate SD from three biological replicates. ***P<0.005, two-tailed Student's t-test. **(C)** TIDE analysis plots (https://tide.deskgen.com) of indel distribution generated by sgRNAs targeting TRAM2 in both MCF10A control and MCF10A-YAP^{5SA} cells. **(D)** The indicated cell populations were subjected to growth competition assays, as detailed in Fig. 3E.

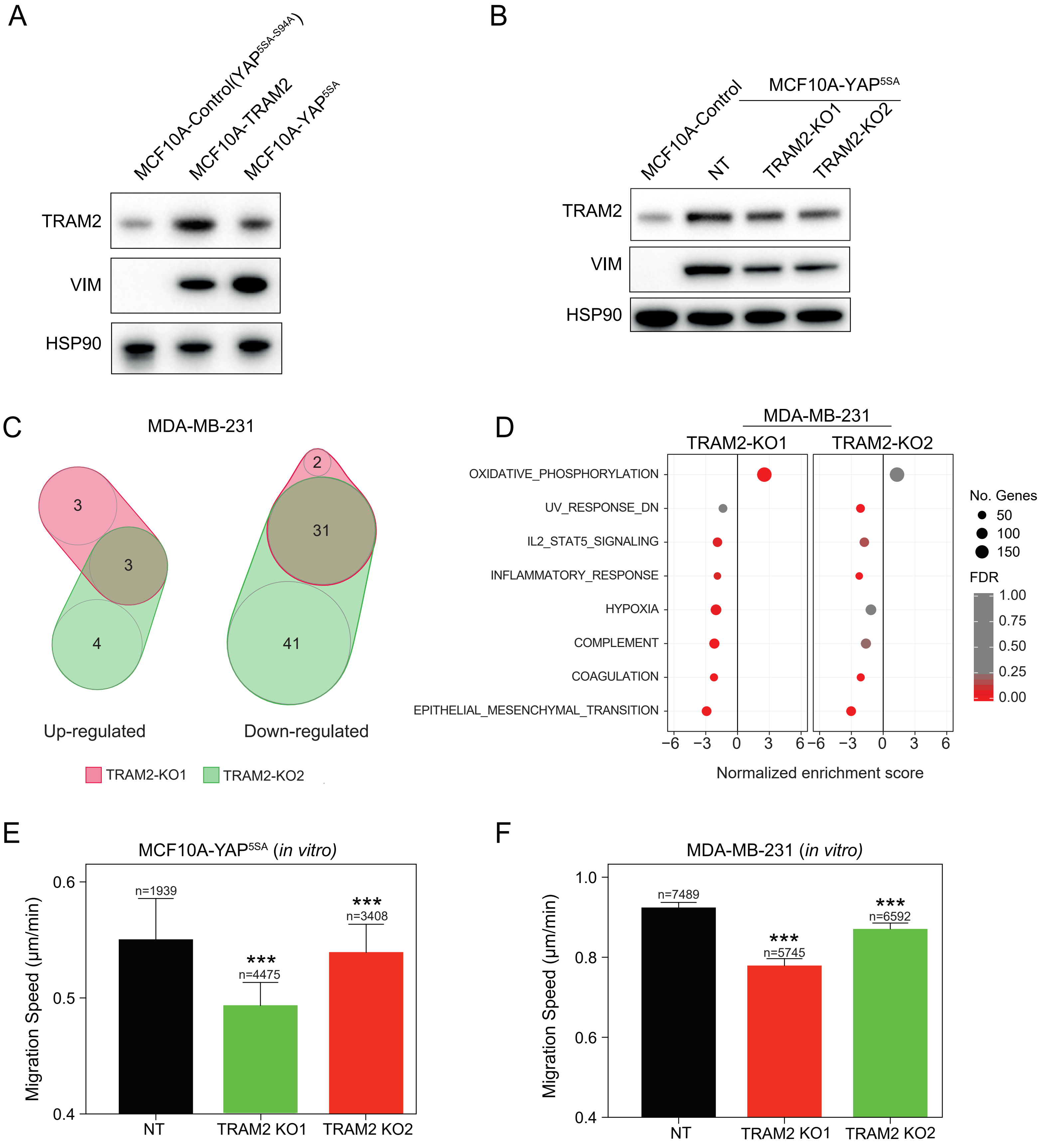

Fig. S6. TRAM2 mediates EMT.
(A,B) Western blot analysis of TRAM2 and VIM levels in MCF10A cells overexpressing TRAM2 and YAP^{5SA}, and in MCF10A-YAP^{5SA} cells with TRAM2-KOs. **(C)** Venn diagrams representing the overlap of up- (p-value: 4.67E-8) or down- (p-value: 7.00E-57) regulated proteins between TRAM2-KO1 and TRAM2-KO2 samples compared with control samples in MDA-MB-231 cells. **(D)** Bubble plot, as detailed in Fig. 4C, representing the gene set enrichment analysis of the proteomics data in MDA-MB-231 cells. **(E,F)** *In vitro* migration assays in MCF10A-YAP^{5SA} and MDA-MB-231 cells transduced with the indicated lentiviral vectors. NT, non-targeting sgRNA control. Error bars indicate SD from counted cells in each condition. ***P<0.005, two-tailed Student's t-test.

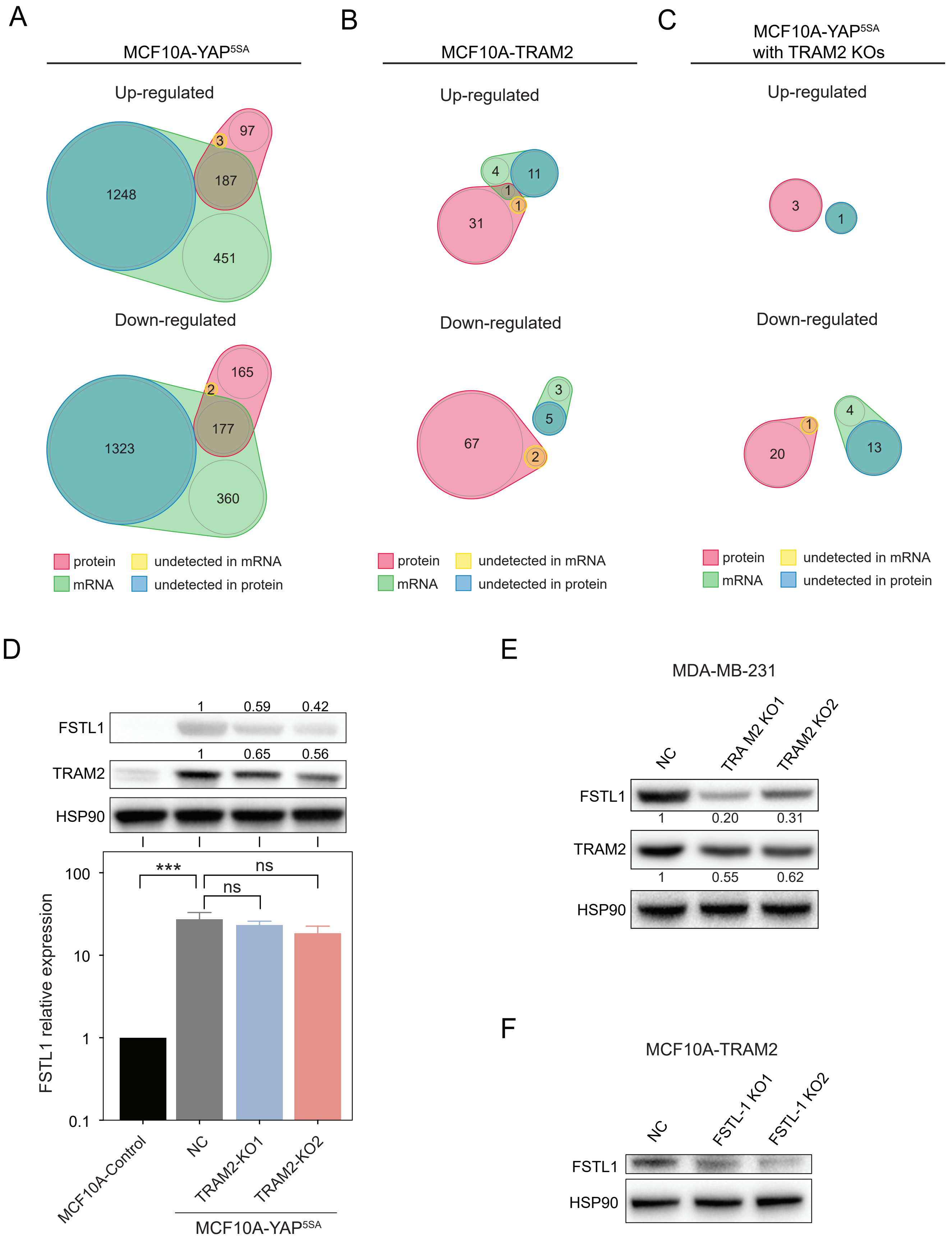

Fig. S7. TRAM2 overexpression and knock-out impacts more at protein level than at RNA level

(A-C) Venn diagrams showing the overlap significantly up- or down-regulated genes between RNA-Seq and proteomics analysis in MCF10A-YAP^{5SA}, MCF10A-TRAM2 cells, and MCF10A-YAP^{5SA} cells with TRAM2-KOs. **(D)** Western blot and qRT-PCR analyses showing FSTL-1 and TRAM2 protein and RNA levels in MCF10A-YAP^{5SA} cells upon TRAM2-KOs. HSP90 was used as loading control. Error bars indicate SD from three biological replicates. ***P<0.005, two-tailed Student's t-test. **(E, F)** Western blot analysis showing FSTL-1 and TRAM2 protein levels in MDA-MB-231 upon knockout of TRAM2 and in MCF10A-TRAM2 cells upon knockout of FSTL-1. HSP90 was used as loading control.
