# Supplementary figures and images for: A comprehensive enhancer screen identifies TRAM2 as a key and novel mediator of YAP oncogenesis
Source: Genome Biol. 2021 Jan 29;22:54. doi: 10.1186/s13059-021-02272-8 (PMC7845134; doi:10.1186/s13059-021-02272-8)

Figure 3B

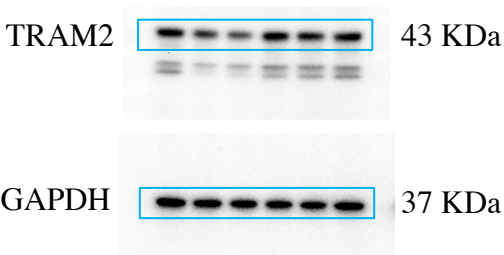

Figure 3F

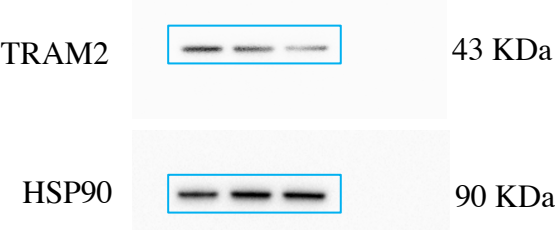

Figure 3H

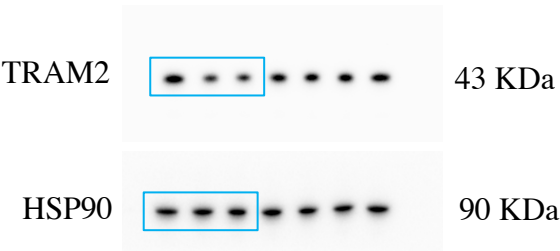

Figure S1A

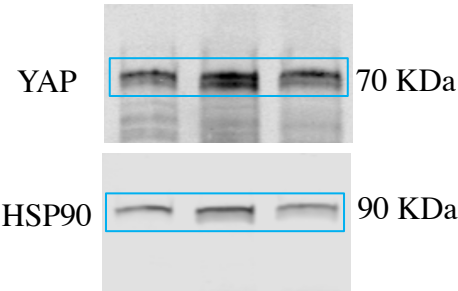

Figure S3B

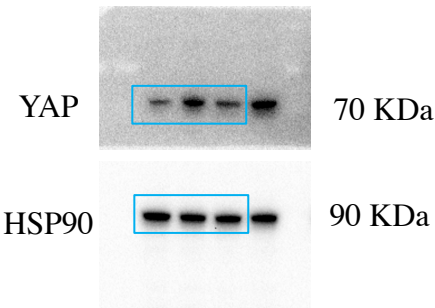

Figure S6A

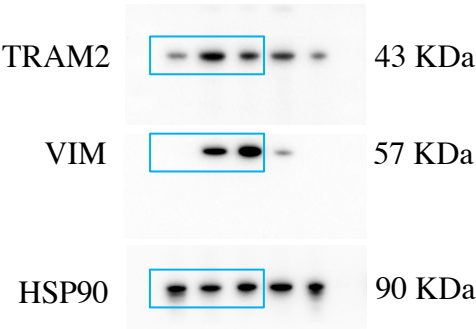

Figure S6B

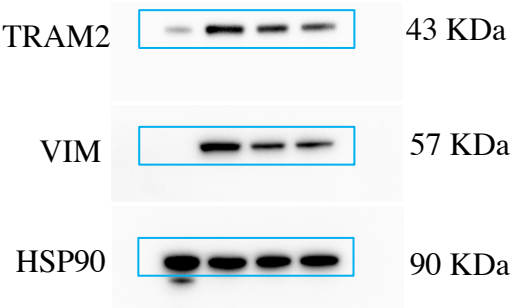

Figure S7D

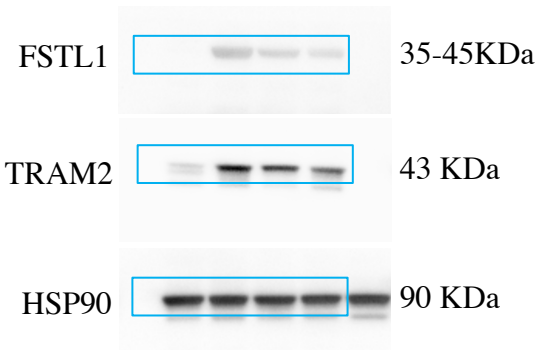

Figure S7E

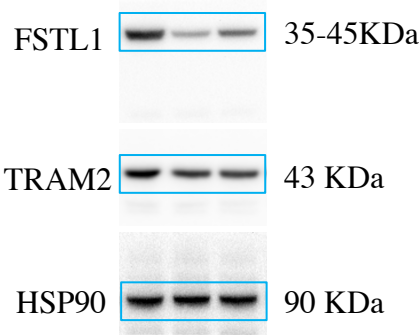

Figure S7F

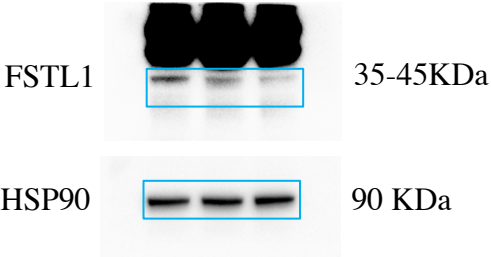

Supplement: Supplementary file 12 — Additional file 12: Complete western blot images of all figures in the manuscript. [file 13059_2021_2272_MOESM12_ESM.pdf]
