# Supplementary material for: A comprehensive enhancer screen identifies TRAM2 as a key and novel mediator of YAP oncogenesis
Source: Genome Biol. 2021 Jan 29;22:54. doi: 10.1186/s13059-021-02272-8 (PMC7845134; doi:10.1186/s13059-021-02272-8)
Supplement: Supplementary file 13 — Additional file 13: Review history. [file 13059_2021_2272_MOESM13_ESM.docx]

Review History

**First round of review**

**Reviewer 1**

**Are you able to assess all statistics in the manuscript, including the appropriateness of statistical tests used?** Yes, and I have assessed the statistics in my report.

**Comments to author:**

This manuscript presents a nice cancer biology and gene regulation study that identifies the role of TRAM2 in YAP oncogenesis. Beginning from enhancer screens, the authors identified a key enhancer of YAP that regulates TRAM2 expression. Follow up experiments on TRAM2 further identified its critical role of TRAM2 in proliferation and migration (via FSTL-1). Overall this is a comprehensive study that combines CRISPR screening, proteomics and other genomics techniques to identify the function of a critical enhancer (and its downstream mechanisms) in oncogenesis.

A few minor comments are listed below.

1. How to select the top ten TEAD4 hits (green dots in Figure 1D). Looks like there are several other grey dots that are similar with the green dots; what is the specific reason to choose green dots but not other grey dots?

2. Expression correlation in Figure 2B. GAPDH is not a suitable negative control since probably GAPDH is not proximal to the enhancer. How about the correlation with other genes near the enhancer but are not among the selected targets?

3. Figure 3C: it's hard to distinguish between different lines (e.g., two green/blue lines). I recommend using different line types to separate groups.

4. Figure 4a: are the p values adjusted for multiple comparisons?

**Reviewer 2**

**Are you able to assess all statistics in the manuscript, including the appropriateness of statistical tests used?** No, I do not feel adequately qualified to assess the statistics.

**Comments to author:**

In this manuscript, Li and colleagues carried out a systematic genomic screening for YAP/TEADs binding target sites (e.g. enhancers), and identify TRAM2 as a mediator of YAP activity, especially in terms of oncogenesis. They employed a wide array of next-generation sequencing methods (Chip-Seq), CRISPR-library screening, bioinformatics, and apply stringent conditions to narrow down their target of interest. They then validate their target using mostly cell-based studies and analysis of cancer patient data.

However, although the manuscript does not lack quality in terms of content per se, the reviewer believes that the presentation of data is too poor for publication in Genome Biology in the current form. Specifically, the reviewer is not at this point not as to the significance of the newfound YAP- TRAM2 axis itself for publication in Genome Biology.

To improve the quality of this manuscript, the following are the comments the reviewer would like to suggest.

1. In Figure 1F, the authors compared changes in luciferase activity of control or enhancer sequence-containing reporter plasmids in YAP overexpressing cells. However, this is problematic for two reasons. First, simply the presence of additional sequences could affect reporter activity, or the transfection efficiency of the different reporter constructs could differ (even though they are normalized to Renilla). Therefore, the authors should compare the reporter activity between MCF10A control and YAP-overexpressing cells.

Secondly, the authors are interested in long-range interactions between enhancer and promoter spanning several mega base pairs, and therefore simply cloning a short region of the enhancer to assess its activity is not only artificial but imprecise. Therefore, if the authors would like to recapitulate the actual conditions, they should clone both the enhancer and promoter sequences separated by at least several kilo base pairs (since it is implausible to clone several mega base pairs), and carry out the experiment as stated in the first concern.

2. In Fig 3B, the authors should present the level of TRAM2 protein in MCF10 cells as a control, thereby its level is truly increased by YAP activation.

3. The authors do not appear to be using single CRISPR/Cas9 knockout clones, which is problematic since the heterogeneity of cells hinders accurate analysis. The incomplete knockout is shown in Figure 3F, H for TRAM2, and Figure S6D for FSTL1. Thus, the reviewer is concerned that the enhancer KO cells used in Figure 3B are heterogenous as well. In the current form, the "KO" cells the authors claim to have generated are no better than si/shRNA-mediated knockdown cells.

4. The survival curve for TRAM2/YAP high/low cancer patients shown in Figure 7A is too shallow to claim a direct relevance in human cancer. The authors should manually confirm this using staining of tissue microarray (TMA) for TRAM2 and YAP to illustrate this point more clearly. There is a fundamental difference in comparing mRNA expression levels based on public gene sets and immunohistochemistry of the proteins themselves, since in many cancers the expression of YAP itself is not altered, but is activated/stabilized by changes at the post-translational level (i.e. dephosphorylation by its upstream kinases, LATS1/2). Therefore, instead of comparing the expression profiles of the only TRAM2 vs. YAP, the authors should compare TRAM2 vs. notable YAP target genes (e.g. CTGF, CYR61, ANKRD1). On a similar note, the authors should overlap the results for Figure S7A and B or display the results for TRAM2 and YAP side-by-side for each TCGA dataset for a clearer representation of the correlation between the two genes.

Lastly, to further highlight the importance of the YAP-TRAM2-FSTL1 axis in actual cancers, it would be meaningful to assess elevated TRAM2/FSTL1 expression or activity in models that have been established to be heavily dependent on YAP, such as K-Ras;p53 mutant.

5. The results presented for FSTL1 being a direct target of TRAM2 (and by extension, YAP) is lacking. Although authors relied on mass spec data to narrow down FSTL1, it would be meaningful to readers to at least validate manually that FSTL1 protein is decreased in TRAM2 or YAP knockout cells, with no changes at its mRNA level.

Moreover, the reviewer feels that the significance of FSTL1 as a downstream target of TRAM2/YAP is inadequately represented. It would be meaningful to see whether FSTL1 KO can alleviate the cancer-associated phenotypes shown in Figure 6E-G in YAP-overexpressing cells, or that overexpression of FSTL1 itself is sufficient to accelerate these phenotypes.

6. It has been well known that YAP/TEADs cooperate with other TFs (Fos, Jun, SRF, etc) to increase the expression of their targets by binding to many enhancers.

Is there any other TFs binding site other than YAP/TEADs in the enhancer region of TRAM2 ?

7. Overall, the data presented in this manuscript are poorly presented and lacks professionalism. The results should be presented in a way that is understandable for the most part in a stand-alone manner, but most figures are not understandable without having to refer to the legends or main text, which significantly disrupts the reading experience. Also, some results are not even presented properly. For example, there are no scale bars for the scratch assay experiments, and there is only an outline for Figure 6E but not Figure 5D. Also, In Figure 5F, no in-figure legends are illustrating what the purple and green staining represents. And, in Figures such as 3E and G, the asterisks representing significance are so tiny such that it is difficult to see (compared to say, Figure 3C in which the asterisks are much bigger). Thus, the reviewer strongly urges the authors to re-organize their data in a much more coherent and legible manner.

8. Not only the data itself, but the wording used throughout the manuscript is also very ambiguous. For example, the authors state "luciferase-reporter assays using a YAP-target region" on page 14 of the main text, but what do the authors mean by this "target region"? They should clarify that this refers to a region of the YAP promoter. The reviewer also noticed many grammatical errors, and therefore highly urges the authors to seek the assistance of a professional editing service.

**Reviewer 3**

**Are you able to assess all statistics in the manuscript, including the appropriateness of statistical tests used?** No, I do not feel adequately qualified to assess the statistics.

**Comments to author:**

In this report, Li et al identified TRAM2, a component of the translocon, as a novel mediator of YAP oncogenesis. The authors carried out a novel genome-wide CRISPR-CAS9-based screen to uncover the functions of YAP-TEAD4 associated enhancers that are required for YAP-driven proliferation using MCF10A cells stably expressing constitutively active (YAP5SA). CRISPR-KOs targeting 7 selected YAP-enhancers in MCF10A-YAP cells revealed potential target genes for 6 out of the 7 YAP-binding enhancers including TRAM2. The group elected to focus on TRAM2 based on the observation of TRAM2 correlation with YAP in various tumor types.

CRISPR-KO targeting the TEAD4 motif in the TRAM2-enhancer region reduced YAP binding to the TRAM2-enhancer region (as assessed by CHIP-qPCR assay) and decreased cell proliferation in MCF10A-YAP cells and the phenotype was rescued by overexpressing TRAM2. In addition, KO of TRAM2 decreased cell proliferation. Additional analysis uncovered EMT signature genes linked with YAP and TRAM2 OE. Overexpression of TRAM2 promoted cell migration and invasiveness but not tumor growth in vivo. The group generated data suggesting that a feedback mechanism or TRAM2 acting upstream of YAP was not involved by performing CHIP-qPCR and luciferase-reporter assays. Presented data suggested that TRAM2 functions at the post-transcriptional level and it was demonstrated that FSTL-1 plays a key role in a TRAM2-dependent EMT phenotype. High TRAM2 expression was shown to correlate with poor survival probability in cancer patients. The authors use a novel approach and identify a new target. The data supporting a role for TRAM2 in cancer is in general supported by the data. However, whether TRAM2 acts through FSTL-1 to modulate YAP in cancers and whether the key observations are recapitulated in cancer cell lines outside of the YAP engineered MCF10A cells remains unclear.

Major comments:

There has been some evidence (unpublished) that expression of YAP5SA in MCF10A cells does not enhance cell proliferation. The way that the authors have plotted cell proliferation throughout the paper, makes it impossible to determine whether the same is true in their cell line. What does the Y-axis "Relative % growth competition" mean? The cells do appear to show an EMT phenotype though (Figure S1) consistent with data in this regard.

Much of the data in the paper is based on analysis of MCF10A cells overexpressing YAP. The manuscript would be significantly enhanced if data linking YAP and TRAM2 in cancer cell lines with active YAP rather than in the YAP mutant engineered line were included. For example, what is the effect of YAP KD or overexpression on TRAM2 mRNA and protein levels, or does treating cells with verteporfin which inhibit YAP-TEAD interaction alter TRAM2 levels?

FSTL-1 is a known YAP target gene (it is within the 50 gene cross-cancer YAP target gene signature reported by the Piccolo lab). How do the authors reconcile this with the claim that FSTL-1 is a post-translational target of TRAM2? Could the observed effects be explained by the fact that FSTL-1 is a YAP target?

Figure S1

The WB showing YAP protein level was missing in Fig. S1. Does TRAM2 expression change in MCF10A-YAP5SA versus MCF10A-YAP5SA-S94A cells by IF or blotting?

Figure 3.

There is a rather moderate effect of enhancerTRAM2-KO on TRAM2 protein level (Fig 3B). Can the authors suggest an explanation? Was the effect on TRAM2 mRNA by qPCR tested? This should be feasible based on RNA seq data, Fig 2B). In light of the modest decrease, how is the strong effect on cell proliferation observed (Fig. 3C)?

Figure 4

Given that the TRAM2 KO efficiency is rather modest in MCF10A-YAP in Fig 4E ((levels shown in WB in Fig 3F) could this explain the relatively low level of overlap?.

The authors may want to consider examining the effect of TRAM2 KD or overexpression on epithelial markers such as E-cadherin and ZO-1 and mesenchymal markers for example vimentin and fibronectin 1 by WB or IF.

Figure 5

The TRAM2 protein levels should be shown for Fig. 5A-B (and other panels such as Fig 6C). Fig S5C-D, the plots indicate rather moderate changes that are discrepant with the high statistical significance markings.

Figure 6

The authors should validate results from the mass spec and RNA-seq data identifying FSTL-1 for example by KO or KD of TRAM2 and examining FSTL-1 mRNA or protein levels.

Data is provided that FSTL-1 acts downstream of TRAM2, but it should be confirmed if FSTL-1 acts downstream of YAP.

Figure 7.

Is it the same patients that have high TRAM2 and high YAP expression and thus display poor survival?

Minor Comments:

Minor point:

It would be helpful for the readers to add that the YAPS94A mutation disrupts YAP-TEAD interaction and also to refer to cells as MCF10A-YAP5SA instead of MCF10A-YAP.

Use better, more accurate wording instead of "effective TRAM2 KO" on Line 42.

**Authors Response**

**Point-by-point responses to the reviewers’ comments:**

Reviewer #1: This manuscript presents a nice cancer biology and gene regulation study that identifies the role of TRAM2 in YAP oncogenesis. Beginning from enhancer screens, the authors identified a key enhancer of YAP that regulates TRAM2 expression. Follow up experiments on TRAM2 further identified its critical role of TRAM2 in proliferation and migration (via FSTL-1). Overall this is a comprehensive study that combines CRISPR screening, proteomics and other genomics techniques to identify the function of a critical enhancer (and its downstream mechanisms) in oncogenesis.

A few minor comments are listed below.

1. How to select the top ten TEAD4 hits (green dots in Figure 1D). Looks like there are several other grey dots that are similar with the green dots; what is the specific reason to choose green dots but not other grey dots?

**Thanks for the comment. We chose the hits based on the parameter that each region has at least 2 different sgRNAs showing the same effect in all three biology replicates. We also set a cutoff (LFC <-0.5) for the effect on MCF10A-YAP and a cutoff (-0.5< LFC <0.5 ) for MCF10A cells in all three biology replicates . The green dots meet these parameters. The Grey dots are the hits that either have only 1 sgRNA per region showing the effect or the LFC from 3 biology replicates are not consistent with LFC cutoff. We have detailed the selection procedure better in the revised manuscript.**

2. Expression correlation in Figure 2B. GAPDH is not a suitable negative control since probably GAPDH is not proximal to the enhancer. How about the correlation with other genes near the enhancer but are not among the selected targets?

**We agree with the reviewer. In the revised manuscript we reanalyzed the data using YAP signature (YAP activity) instead of YAP mRNA levels (as recommended by Reviewer #2 below), and therefore omitted the use of GAPDH mRNA as negative control. We observed that our selected targeted genes all correlate with YAP signature in most of the tumor types (Figure 2B). The flanking genes showed little correlation or were not expressed in most cancer types (Figure S4B).**

3. Figure 3C: it's hard to distinguish between different lines (e.g., two green/blue lines). I recommend using different line types to separate groups.

**Thanks for the comment. We changed the lines color accordingly.**

4. Figure 4a: are the p values adjusted for multiple comparisons?

**Thanks for the comment. We did not correct for multiple testing. We agree that it is a common procedure for large datasets. However, global analyses like GSEA are not affected by multiple testing corrections, since genes are ranked by a metric based on the p-value and the sign of the change. Doing multiple test correction will not affect the ranking of the genes using this metric (if protein A has lower p-value than protein B, it will also have a lower adjusted p-value than protein B). Regarding target selection, we agree that multiple test correction would decrease the false discovery rate, but at the cost of maybe missing important findings (see, for example, “No adjustments are needed for multiple comparisons. K J Rothman.** [1]**. We think that intersecting 4 conditions (each of them with triplicates) using raw p-values constitutes a good balance between type I (false positive) and type II error (false negative). Importantly, no conclusions have been made without validating the results independently using western-blot.**

Reviewer #2: In this manuscript, Li and colleagues carried out a systematic genomic screening for YAP/TEADs binding target sites (e.g. enhancers), and identify TRAM2 as a mediator of YAP activity, especially in terms of oncogenesis. They employed a wide array of next-generation sequencing methods (Chip-Seq), CRISPR-library screening, bioinformatics, and apply stringent conditions to narrow down their target of interest. They then validate their target using mostly cell-based studies and analysis of cancer patient data.

However, although the manuscript does not lack quality in terms of content per se, the reviewer believes that the presentation of data is too poor for publication in Genome Biology in the current form. Specifically, the reviewer is not at this point not as to the significance of the newfound YAP- TRAM2 axis itself for publication in Genome Biology.

To improve the quality of this manuscript, the following are the comments the reviewer would like to suggest.

1. In Figure 1F, the authors compared changes in luciferase activity of control or enhancer sequence-containing reporter plasmids in YAP overexpressing cells. However, this is problematic for two reasons. First, simply the presence of additional sequences could affect reporter activity, or the transfection efficiency of the different reporter constructs could differ (even though they are normalized to Renilla). Therefore, the authors should compare the reporter activity between MCF10A control and YAP-overexpressing cells.

**We appreciate this comment. We indeed performed the luciferase assays with MCF10A and MCF7 cell lines (both cell lines have very low YAP activity) at the same time as the MCF10A-YAP^5SA^ cells. However, we realized that in contrast to MCF7, MCF10A cells are very difficult to transfect, resulting in inconsistent luciferase signals that we cannot use for publication. Therefore, we included instead the luciferase assay data of MCF7 cells. The result is presented below for the reviewer. (***P<0.005, **P<0.01, *P<0.05, two-tailed Student’s t-test). We observed that MCF10A-YAP^5SA^ cells generally showed higher enhancer activity compared to MCF7 cells, with the exception of Enhancer C that showed strong activity in MCF7 cells. This result can be explained by the characterized ERα-binding site within Enhancer C which in MCF7 cells was shown to regulate CCND1 expression**[2] **(Korkmaz G, Lopes R, Ugalde AP, Nevedomskaya E, Han R, Myacheva K, et al. Functional genetic screens for enhancer elements in the human genome using CRISPR-Cas9. Nat Biotechnol. 2016).**

Secondly, the authors are interested in long-range interactions between enhancer and promoter spanning several mega base pairs, and therefore simply cloning a short region of the enhancer to assess its activity is not only artificial but imprecise. Therefore, if the authors would like to recapitulate the actual conditions, they should clone both the enhancer and promoter sequences separated by at least several kilo base pairs (since it is implausible to clone several mega base pairs), and carry out the experiment as stated in the first concern.

**We respectfully disagree with the opinion of the reviewer on this point. This Type of experiment is widely used to uncover promoters and enhancers in the human genome. We placed the cloned region downstream of the reporter gene, such that indeed it is distant by several kbs from the promoter. We do agree that this assay is only an indication for enhancer activity, and comes in addition to the other evidence described in the paper (3D chromatin interaction and histone marks).**

2. In Fig 3B, the authors should present the level of TRAM2 protein in MCF10 cells as a control, thereby its level is truly increased by YAP activation.

**Thanks for the comment. We added an immunoblot analysis that compares the levels of TRAM2 in MCF10A and MCF10A-YAP cells. (Figure S6A, S6B).**

3. The authors do not appear to be using single CRISPR/Cas9 knockout clones, which is problematic since the heterogeneity of cells hinders accurate analysis. The incomplete knockout is shown in Figure 3F, H for TRAM2, and Figure S6D for FSTL1. Thus, the reviewer is concerned that the enhancer KO cells used in Figure 3B are heterogenous as well. In the current form, the "KO" cells the authors claim to have generated are no better than si/shRNA-mediated knockdown cells.

**We respectfully disagree with the opinion of the reviewer on this point. We chose to use polyclonal CRISPR-KO populations for validations, as we believe these are more reliable to reflect the real effect on the function of the targeted genes. From our vast past experience, single CRISPR clones are likely to behave in different manners due to slightly different genomic/epigenomic backgrounds. This is mostly negated when looking at polyclonal populations. Moreover, in contrast to si/shRNAs where cells have variable levels of suppression, CRISPR-KOs can generate complete KO in most of the cells in the population. The remaining signal is due to half or no KO at all of few cells. In addition, the remaining signal isn’t necessarily functional protein, since it may contain mutations and still be detected by WB.**

4. The survival curve for TRAM2/YAP high/low cancer patients shown in Figure 7A is too shallow to claim a direct relevance in human cancer. The authors should manually confirm this using staining of tissue microarray (TMA) for TRAM2 and YAP to illustrate this point more clearly. There is a fundamental difference in comparing mRNA expression levels based on public gene sets and immunohistochemistry of the proteins themselves, since in many cancers the expression of YAP itself is not altered, but is activated/stabilized by changes at the post-translational level (i.e. dephosphorylation by its upstream kinases, LATS1/2). Therefore, instead of comparing the expression profiles of the only TRAM2 vs. YAP, the authors should compare TRAM2 vs. notable YAP target genes (e.g. CTGF, CYR61, ANKRD1). On a similar note, the authors should overlap the results for Figure S7A and B or display the results for TRAM2 and YAP side-by-side for each TCGA dataset for a clearer representation of the correlation between the two genes.

**Thanks for this comment, it has really helped us improve our manuscript. While performing IHQ of TRAM2 and YAP in tumor samples would be more conclusive, it is a long time-consuming experiment that would exceed the time limit for this revision. However, we agree completely with the reviewer that YAP mRNA is not a good proxy of YAP activity. Instead, and as mentioned in another comment, we used a YAP 57-gene signature defined by Piccolo lab** [3]**(The Hippo transducer TAZ confers cancer stem cell-related traits on breast cancer cells, 2011, Cordenonsi , S,Piccolo S) to score samples for YAP activity and perform survival analysis using this metric. As expected (and described in “Comprehensive Molecular Characterization of the Hippo Signaling Pathway in Cancer. Wang et al, Cell Reports 2018”, for a shorter YAP gene signature), the YAP gene signature score correlates significantly with poor survival in 13 TCGA studies. In line with TRAM2 being a bona-fide target of YAP, TRAM2 expression alone predicts poor survival in 8 TCGA studies, of which 6 of them (75%) are also significant using the YAP gene signature score and yield similar survival curves (Figure 7A and 7B). Following reviewer suggestions, we have replaced the previous Figure S7A and S7B with a more comprehensive figure that summarizes the results of Cox regression analysis for TRAM2 expression and YAP gene signature side by side using a heatmap-like plot (Figure 7A in the revised version). TCGA studies where survival is significantly associated with both TRAM2 expression and YAP gene signature score have been plotted side by side using Kaplan-Meier plots (Figure 7B in the revised manuscript). Of note, that following your suggestion for survival analysis, we have also used the YAP gene signature score instead of YAP mRNA to perform the gene expression correlations of selected YAP target genes in figure 2B.**

Lastly, to further highlight the importance of the YAP-TRAM2-FSTL1 axis in actual cancers, it would be meaningful to assess elevated TRAM2/FSTL1 expression or activity in models that have been established to be heavily dependent on YAP, such as K-Ras;p53 mutant.

**Thanks for the comment.  The function of FSTL1 is out of the scope of this manuscript. Our manuscript focuses on enhancer screens, YAP and TRAM2. We use FSTL1 only to pinpoint TRAM2 role.**

5. The results presented for FSTL1 being a direct target of TRAM2 (and by extension, YAP) is lacking. Although authors relied on mass spec data to narrow down FSTL1, it would be meaningful to readers to at least validate manually that FSTL1 protein is decreased in TRAM2 or YAP knockout cells, with no changes at its mRNA level.

**In our analysis we compared protein levels (by mass spec) and mRNA levels (by RNAseq). These are the best method so far for measuring differential expression at the protein and mRNA levels, as they rely on proteomic and genomic data analyses, removing doubts about control refence gene expression. In the revised manuscript, as requested by reviewer, we validated that FSTL1 protein expression decreased upon TRAM2-KO in MCF10A-YAP^5SA^ cells, with no changes at its mRNA level. This data is in line with our proteomics and RNAseq data. (Figure S7D in the revised manuscript).**

Moreover, the reviewer feels that the significance of FSTL1 as a downstream target of TRAM2/YAP is inadequately represented. It would be meaningful to see whether FSTL1 KO can alleviate the cancer-associated phenotypes shown in Figure 6E-G in YAP-overexpressing cells, or that overexpression of FSTL1 itself is sufficient to accelerate these phenotypes.

**Thanks for this comment. Our manuscript focused on enhancer screens, YAP and TRAM2, and we believe that the role of FSTL1 in cancer is out of the scope. Moreover, the function of FSTL1 in migration and cancer metastasis was investigated before** [4][5]**.**

6. It has been well known that YAP/TEADs cooperate with other TFs (Fos, Jun, SRF, etc) to increase the expression of their targets by binding to many enhancers.

Is there any other TFs binding site other than YAP/TEADs in the enhancer region of TRAM2 ?

**Thanks for this comment. It is not surprising that the enhancer of TRAM2 contains many potential transcription-factor binding sites other than YAP. Most interestingly, there is an AP1 (FOS/JUN) binding site in this region. The group of Picollo has already reported in an elegant paper**[6] **that AP1 can collaborate with YAP/TEAD4 to enhance oncogenic growth. Indeed, we also observed that there is JUN binding peak close to the YAP/TEAD4 binding peak in Piccolo’s Chip-seq data sets. Whether this AP1 site contributes to YAP-TRAM2 axis will require further investigation, but is out of the scope of this manuscript.**

7. Overall, the data presented in this manuscript are poorly presented and lacks professionalism. The results should be presented in a way that is understandable for the most part in a stand-alone manner, but most figures are not understandable without having to refer to the legends or main text, which significantly disrupts the reading experience. Also, some results are not even presented properly. For example, there are no scale bars for the scratch assay experiments, and there is only an outline for Figure 6E but not Figure 5D. Also, In Figure 5F, no in-figure legends are illustrating what the purple and green staining represents. And, in Figures such as 3E and G, the asterisks representing significance are so tiny such that it is difficult to see (compared to say, Figure 3C in which the asterisks are much bigger). Thus, the reviewer strongly urges the authors to re-organize their data in a much more coherent and legible manner.

**We thank the reviewer for pinpointing these errors in the manuscript, and changed asterisks size, added the scale bars and outline for the migration assay presented in Figure 5D. Further, we also added details to the legends as requested.**

8. Not only the data itself, but the wording used throughout the manuscript is also very ambiguous. For example, the authors state "luciferase-reporter assays using a YAP-target region" on page 14 of the main text, but what do the authors mean by this "target region"? They should clarify that this refers to a region of the YAP promoter. The reviewer also noticed many grammatical errors, and therefore highly urges the authors to seek the assistance of a professional editing service.

**In the original corresponding Figure legend, we already explained that “the indicated cell populations were subjected to luciferase assays using Enhancer E”. The YAP-target region that we used is therefore Enhancer E. We now included this information also into the main text in the revised manuscript to prevent ambiguity. We also corrected remaining grammar errors in the manuscript.**

Reviewer #3: In this report, Li et al identified TRAM2, a component of the translocon, as a novel mediator of YAP oncogenesis. The authors carried out a novel genome-wide CRISPR-CAS9-based screen to uncover the functions of YAP-TEAD4 associated enhancers that are required for YAP-driven proliferation using MCF10A cells stably expressing constitutively active (YAP5SA). CRISPR-KOs targeting 7 selected YAP-enhancers in MCF10A-YAP cells revealed potential target genes for 6 out of the 7 YAP-binding enhancers including TRAM2. The group elected to focus on TRAM2 based on the observation of TRAM2 correlation with YAP in various tumor types.

CRISPR-KO targeting the TEAD4 motif in the TRAM2-enhancer region reduced YAP binding to the TRAM2-enhancer region (as assessed by CHIP-qPCR assay) and decreased cell proliferation in MCF10A-YAP cells and the phenotype was rescued by overexpressing TRAM2. In addition, KO of TRAM2 decreased cell proliferation. Additional analysis uncovered EMT signature genes linked with YAP and TRAM2 OE. Overexpression of TRAM2 promoted cell migration and invasiveness but not tumor growth in vivo. The group generated data suggesting that a feedback mechanism or TRAM2 acting upstream of YAP was not involved by performing CHIP-qPCR and luciferase-reporter assays. Presented data suggested that TRAM2 functions at the post-transcriptional level and it was demonstrated that FSTL-1 plays a key role in a TRAM2-dependent EMT phenotype. High TRAM2 expression was shown to correlate with poor survival probability in cancer patients. The authors use a novel approach and identify a new target. The data supporting a role for TRAM2 in cancer is in general supported by the data. However, whether TRAM2 acts through FSTL-1 to modulate YAP in cancers and whether the key observations are recapitulated in cancer cell lines outside of the YAP engineered MCF10A cells remains unclear.

Major comments:

There has been some evidence (unpublished) that expression of YAP5SA in MCF10A cells does not enhance cell proliferation. The way that the authors have plotted cell proliferation throughout the paper, makes it impossible to determine whether the same is true in their cell line. What does the Y-axis "Relative % growth competition" mean? The cells do appear to show an EMT phenotype though (Figure S1) consistent with data in this regard.

**We thank the reviewer for this interesting comment. We observed that the expression of YAP^5SA^ in MCF10A cells does not accelerate cell proliferation when supplemented with EGF, but the expression of YAP^5SA^ in MCF10A cells does have growth advantage without EGF (Figure S1B, up panel). This is in line with previous observations**[7]**. For clarity, we changed the Y-axis into relative % of MCF10A cells (upper panel) and the Y-axis into relative % of YAP^5SA^-sgRNA cells (lower panel).**

Much of the data in the paper is based on analysis of MCF10A cells overexpressing YAP. The manuscript would be significantly enhanced if data linking YAP and TRAM2 in cancer cell lines with active YAP rather than in the YAP mutant engineered line were included. For example, what is the effect of YAP KD or overexpression on TRAM2 mRNA and protein levels, or does treating cells with verteporfin which inhibit YAP-TEAD interaction alter TRAM2 levels?

**We thank the reviewer for this insightful comment. We already validated several key findings with MDA-231 cell line (known to have high YAP activity) in our previous manuscript. In the revised manuscript we additionally examined TRAM2 expression in MCF10A-YAP^5SA^ cell lines treated with verteporfin (2uM). We included CCND1 as an internal positive control to verify the effect on TRAM2 expression by inhibiting YAP-TEAD interaction. As expected, CCND1 expression was decreased upon verteporfin treatment. Concerning TRAM2, we also observed reduced expression in treated cells, indicating that YAP-TEAD interaction is required for TRAM2 activation (Figure S5B in the revised manuscript)**

FSTL-1 is a known YAP target gene (it is within the 50 gene cross-cancer YAP target gene signature reported by the Piccolo lab). How do the authors reconcile this with the claim that FSTL-1 is a post-translational target of TRAM2? Could the observed effects be explained by the fact that FSTL-1 is a YAP target?

**We thank the reviewer for this point. In our datasets we observed an increased mRNA level of FSTL1 in MCF10A-YAP^5SA^ cells, but not in the MCF10A-TRAM2 cells. However, at the protein level, we observed increased FSTL1 protein level in both MCF10A-YAP^5SA^ cells and MCF10A-TRAM2 cells (Figure 4A, 4B, 6C, 6D). This indicates the contribution of TRAM2 to FSTL1 expression. In addition, TRAM2-KO in MCF10A-YAP^5SA^ cells decreased FSTL1 protein level without affecting FSTL1 RNA level (Figure 4E, 4F, 6C, 6D and S7D). TRAM2 is a component of the translocon that recognizes the signal peptide of some newly synthesized proteins. FSTL1 has a strong signal peptide motif, leading us to conclude that FSTL1 is a post-translational target of TRAM2. We believe that different cell types may behave differently in response to YAP, and that the expression of co-transcriptional factors may cause differences in YAP transcription response. Nevertheless, the fact that FSTL1 can be either a direct target of YAP in some cell types, or post-translational target via TRAM2, or both, strengthens the connection between YAP, TRAM2, FSTL1, and EMT phenotype.**

Figure S1

The WB showing YAP protein level was missing in Fig. S1. Does TRAM2 expression change in MCF10A-YAP5SA versus MCF10A-YAP5SA-S94A cells by IF or blotting?

**We thank the reviewer for this point. In the revised manuscript, we incorporated YAP protein levels into Figure S1A. We measured the TRAM2 expression in MCF10A-YAP^5SA^ and MCF10A-YAP^5SA-S94A^ cells including the positive control MCF10A-TRAM2 cells (Figure S6A). As predicted, we observed higher TRAM2 expression in MCF10A-YAP^5SA^ compared with MCF10A-Control (YAP^5SA-S94A^) Cells.**

Figure 3.

There is a rather moderate effect of enhancerTRAM2-KO on TRAM2 protein level (Fig 3B). Can the authors suggest an explanation? Was the effect on TRAM2 mRNA by qPCR tested? This should be feasible based on RNA seq data, Fig 2B). In light of the modest decrease, how is the strong effect on cell proliferation observed (Fig. 3C)?

**We appreciate this comment. It is well established that knocking out an enhancer will only affect the activation of its target gene, without affecting its basal level. In addition, we used a polyclonal population of enhancer TRAM2-KO cells which may give rise to a more moderate effect than a selected clone. These two reasons are likely explanation to a moderate 30-40% reduction in TRAM2 protein level upon enhancer TRAM2-KO.**

**Instead of using qPCR to test the effect of Enhancer-TRAM2-KO on TRAM2 mRNA, we used RNA-seq which we believe is a more accurate and less sensitive method (Figure 2A, Table S4).**

**Lastly, all the selected enhancers-KOs - including enhancer TRAM2-KO - showed a moderate proliferation decrease (a drop to 70%-80%, Figure 1G, Figure 3C) compared to YAP-KO control (which reduced proliferation to ~30%, Figure 1G) after 12 days. We therefore concluded in our manuscript that YAP regulates cell proliferation through several enhancers and their target genes, each of which with a relatively moderate contribution.**

Figure 4

Given that the TRAM2 KO efficiency is rather modest in MCF10A-YAP in Fig 4E ((levels shown in WB in Fig 3F) could this explain the relatively low level of overlap?.

**Thank for this comment. We decided to use Venn diagrams (Figure 4F) to be consistent through the paper, as Venn diagrams are useful to show overlap between different conditions. However, we calculated the p value of the intersection using fisher exact test, which yielded significant overlap between two KOs as indicated in the figure legends (Venn diagrams representing the overlap of up- (p-value: 9.193718E-08) or down-(p-value: 7.649084E-58) regulated proteins between TRAM2-KO1 and TRAM2-KO2 samples compared with control in Panel E.). The caveat of intersecting significant protein lists between two samples using arbitrary cutoffs based on p values and fold changes is that if the KO efficiency is slightly different, some proteins might not pass the filtering while they still correlate well. Thus, we performed a scatter plot to show the correlation between KO1 and KO2 as shown below. This yielded very significant correlation between the two KOs.**

The authors may want to consider examining the effect of TRAM2 KD or overexpression on epithelial markers such as E-cadherin and ZO-1 and mesenchymal markers for example vimentin and fibronectin 1 by WB or IF.

**We thank the reviewer for this point. In the revised manuscript, we measured the VIM expression with TRAM2 KD and overexpression. In line with our proteomic data, we observed that VIM was up-regulated upon TRAM2 overexpression in MCF10A cells (Figure S6A) and down-regulated upon TRAM2-KO in MCF10A-YAP5SA cells (Figure S6B).**

Figure 5

The TRAM2 protein levels should be shown for Fig. 5A-B (and other panels such as Fig 6C). Fig S5C-D, the plots indicate rather moderate changes that are discrepant with the high statistical significance markings.

**We apologize for not including immunoblots of the TRAM2 levels. We now added TRAM2 protein levels to Fig5A and Figure 6C (please see Figure S6A, S6B).**

**We now added the TRAM2 protein levels for Figure 5B as below. However, we do not see a big difference of TRAM2 expression in MDA-231-TRAM2 overexpressing cells compared to MDA-231 control cells by WB. This can be explained by the limitation of WB detection since the signal seems saturated due to high basal TRAM2 expression in MDA-231 cells. To solidify the overexpression of TRAM2 in MDA-231 cells, we performed mRNAseq analysis, which is more quantitative than immunoblot analysis, and observed a clear elevated TRAM2 levels in MDA-231-TRAM2 cells compared to control cells as shown below.**

**For Fig S5C-D, the difference between the means of the experimental and control groups is larger than multiple folds of SEM, which is the reason why we get a statistically significant result although the change is moderate. The reason for a low SEM is due to the large sample size (thousands of cells) we counted.**

Figure 6

The authors should validate results from the mass spec and RNA-seq data identifying FSTL-1 for example by KO or KD of TRAM2 and examining FSTL-1 mRNA or protein levels.

Data is provided that FSTL-1 acts downstream of TRAM2, but it should be confirmed if FSTL-1 acts downstream of YAP.

**We thank the reviewer for this point. In the revised manuscript, we validated the effect of TRAM2-KO in MCF10A-YAP^5SA^ cells on FSTL-1 mRNA and protein levels (Figure S7D).**

Figure 7.

Is it the same patients that have high TRAM2 and high YAP expression and thus display poor survival?

**We thank the reviewer for this point. In the revised manuscript, we have included extra analyses based on comments from reviewer 2. We have now used a YAP gene signature score as a proxy of YAP activity (instead of YAP mRNA) to perform survival analysis (Figure 7A and B in the revised manuscript) and for gene expression correlations of selected YAP target genes (Figure 2B). Given the high expression correlation between TRAM2 and the YAP gene signature score in most TCGA studies, it is expected that TRAM2 will be highly expressed in patients with high YAP score. However, following your suggestion, we have included a plot that shows TRAM2 expression in patients that were classified as high or low for the YAP gene signature score in TCGA studies that yielded significant survival analysis results (Figure 7C in the revised manuscript). In all cases, Wilcoxon rank sum test gave highly significant differences in TRAM2 expression between low and high YAP gene signature score groups.**

Minor Comments:

Minor point:

It would be helpful for the readers to add that the YAPS94A mutation disrupts YAP-TEAD interaction and also to refer to cells as MCF10A-YAP5SA instead of MCF10A-YAP.

**We thank the reviewer for this point. In the revised manuscript, we indicated that YAP^S94A^ mutation disrupts YAP-TEAD interaction. We also refer to cells as MCF10A-YAP^5SA^ instead of MCF10A-YAP through all the manuscript including figures.**

Use better, more accurate wording instead of "effective TRAM2 KO" on Line 42.

**We thank the reviewer for this point. In the revised manuscript, we deleted the word “effective”.**

**References**

1. Rothman KJ. No adjustments are needed for multiple comparisons. Epidemiology. 1990;

2. Korkmaz G, Lopes R, Ugalde AP, Nevedomskaya E, Han R, Myacheva K, et al. Functional genetic screens for enhancer elements in the human genome using CRISPR-Cas9. Nat Biotechnol. 2016;

3. Cordenonsi M, Zanconato F, Azzolin L, Forcato M, Rosato A, Frasson C, et al. The hippo transducer TAZ confers cancer stem cell-related traits on breast cancer cells. Cell. 2011;

4. Gu C, Wang X, Long T, Wang X, Zhong Y, Ma Y, et al. FSTL1 interacts with VIM and promotes colorectal cancer metastasis via activating the focal adhesion signalling pathway. Cell Death Dis. 2018;

5. Chi-Chung Lau M, Ng KY, Wong TL, Tong M, Lee TK, Ming XY, et al. FSTL1 promotes metastasis and chemoresistance in esophageal squamous cell carcinoma through NFκB-BMP signaling cross-talk. Cancer Res. 2017;

6. Zanconato F, Forcato M, Battilana G, Azzolin L, Quaranta E, Bodega B, et al. Genome-wide association between YAP/TAZ/TEAD and AP-1 at enhancers drives oncogenic growth. Nat Cell Biol. 2015;

7. Overholtzer M, Zhang J, Smolen GA, Muir B, Li W, Sgroi DC, et al. Transforming properties of YAP, a candidate oncogene on the chromosome 11q22 amplicon. Proc Natl Acad Sci. 2006;

**Second round of review**

**Reviewer 1**

There's one concern in related to the issue #4 on figure 4a. It's uncommon to use raw p values rather than adjusted p values for large datasets like in figure 4a. If there are triplicates in each condition, it would definitely be a concern if no significant hits are found (after p value adjustment). From the method description, t-test was used for figure 4a. This may be the reason for low statistical power in figure 4a, since in this case t-test only use 6 data points. I would recommend using other statistical methods for differential analysis (like edgeR or DESeq2), since they will have much better statistical power than t-test.

**Reviewer 3**

Comments regarding the response to Reviewer #2.

Many of the comments have been adequately addressed with the exception of the following:

Comment 1: In Figure 1F. Comparing reporter activation in MCF7 cells to MCF10A-YAP5SA is not a valid comparison. While comparison to MCF10A would have been better, using the promotor control (as provided in the main paper figure) would normally be acceptable.

However, there is a caveat regarding the reporter construct.

The authors indicate they subcloned the enhancer downstream of the luciferase reporter gene, presumably either up or downstream of the poly-A tail region, rather than downstream of the promoter (ie upstream of the luciferase gene) as I would have expected. Whether this enhancer placement is considered appropriate needs be confirmed by an expert. If what was meant was that the enhancer is downstream of the promoter (but upstream of the reporter) then an equivalent ‘control’ DNA fragment should have been used in the control construct, as the distance of the promoter from gene start can significantly impact transcription. If this is not available/not tested, then only alternative, is to directly compare parental and YAP5SA constructs in the SAME cell line (ie refer to point above).

Comment 3: Regarding using a population rather than clones of the Crispr KO: the rationale presented by the authors that a pool is better than clones would be true in cases of efficient knockdown, but in contrast to their claim that only a few cells in the pool do not display KO, the blots show a reduction of only 40-60% of control protein levels, suggesting about half the cells do not have a KO. This heterogeneity (ie an estimated 50% of cells in the pool display KO), must be mentioned in the text.

Considering this KO efficiency, I was initially puzzled to see that the authors show almost complete inhibition of growth (Fig 3E) in the heterogeneous population. However, it became apparent that a truncated Y-axis was used (ie it did not start at zero), creating a misleading impression of the results. This should be corrected in this and all other plots.

Comment 5: Regarding, the request to confirm that there is a reduction of FSTL1 protein but not mRNA levels (Fig S7D). First, I disagree with the author’s response that large scale experiments (Mass Spec and RNAseq) are sufficient for demonstrating differential expression. The authors provide a panel to address this, but the reduction in FSTL1 protein is extremely modest and while the mRNA does not show any change, the authors neglected to confirm the KO efficiency in the same sample, which presumably is using a pool as above). Since pools can change depending on culture times, the absence of this control is particularly problematic.

Comment 5 (continued). The authors response to the comment “Moreover, the reviewer feels that the significance of FSTL1 as a downstream target of TRAM2/YAP is inadequately represented. It would be meaningful to see whether FSTL1 KO can alleviate the cancer-associated phenotypes shown in Figure 6E-G in YAP-overexpressing cells, or that overexpression of FSTL1 itself is sufficient to accelerate these phenotypes.” is that this is beyond the scope of the manuscript.

While, I agree with the authors that so many experiments to support the role of FSTL1 are not required, the abstract states “Mechanistically, we identified FSTL-1 as a major direct client of TRAM2 that is involved in these phenotypes.” Thus, data supporting this statement is required or the statement must be modified. There is only one figure that addresses this comment, Fig. 6G on migration, but the control line, ie non-TRAM2 expressing cells, was not included so there is no evidence to support a mechanistic link between TRAM2 and FSTL1, rather it can only be concluded that FSTL1 can control migration.

Comments regarding the response to Reviewer #3.

The reviewer commends the authors for addressing the majority of questions. However, the following issues remain outstanding. The following suggestions are recommended to improve the final version of the manuscript.

The request to provide data linking YAP and TRAM2 in cancer cell lines with active YAP rather than only in the YAP mutant engineered line was not provided (ie such as YAP KD or overexpression on TRAM2 mRNA/protein levels). While the authors state “We already validated several key findings with MDA-231 cell line”, it was not indicated where this could be found. I was unable to locate this data with the exception of the proliferation data in Fig. S6F, which shows a marginal effect. It should be noted that results derived from an analysis of screens without subsequent validation is not satisfactory.

Figure S1: There is no to very moderate increase in YAP expression level in MCF10A-5SA cells vs control (Figure S1A). The authors may want to consider adding YAP localization by IF to validate the point that the observed phenotypic effect is due to the nuclear localization of YAP5SA and not dependent on the total protein level.

Figure 3. The author's response to the reviewer's comments are unsatisfactory.

I strongly disagree that RNA-seq data does not have to be confirmed. It would be simple to check TRAM2 mRNA levels by qPCR. This is much more quantitative than immunoblotting.

The conclusions regarding the effect of eTRAM2 KO on cell proliferation, would have been substantially stronger if the authors used multiple verified clones rather than a heterogeneous pool of cells. However, this issue alone would not preclude publication.

Figure 5. The TRAM2 expression blot is overexposed. The authors should determine if there is a difference in the TRAM2 protein levels with a lower amount of protein lysate to overcome the saturation issue.

Figure 6. In the new data Figure S7D, there is a very moderate effect of TRAM2-KO in MCF10A-YAP5SA cells on FSTL-1 protein level. The authors should consider either validating by using single clones or determining the impact of TRAM2 Knockout/knockdown on FSTL-1 using a cancer cell line with active YAP such as MDA-MB231 cells.

**Authors Response**

**Point-by-point responses to the reviewers’ comments:**

        Reviewer #1: There's one concern in related to the issue #4 on figure 4a. It's uncommon to use raw p values rather than adjusted p values for large datasets like in figure 4a. If there are triplicates in each condition, it would definitely be a concern if no significant hits are found (after p value adjustment). From the method description, t-test was used for figure 4a. This may be the reason for low statistical power in figure 4a, since in this case t-test only use 6 data points. I would recommend using other statistical methods for differential analysis (like edgeR or DESeq2), since they will have much better statistical power than t-test.

We appreciate this comment. We have addressed this point by performing moderate t-statistics using an empirical Bayes method from limma R-package (Ritchie ME et al, NAR 2015) combined with multiple test correction using Benjamini-Hochberg procedure. The new adjusted p-values are similar to the raw ordinary t-statistics of our previous version, but we agree with the reviewer that this is a more elegant procedure. Since the proteomics data is central in our work, many figures have been updated employing the adjusted p-values this time. This includes all the volcano plots, the GSEA analyses, the Venn diagrams and the TRAM2 target discovery. The updated plots look very similar with slight variations but showing the same message as the previous version in all cases. Please note that given that multiple test correction was employed, we have slightly relaxed the cutoff for differential expression from |LFQ difference| > 0.7 in the previous version to |log2 fold-change| > 0.5. However, please note that for TRAM2 target identification, we still used the raw p-values (and therefore a higher log2 fold-change cutoff of |0.7|), since using the adjusted p-values yields an empty intersection between YAP-OE and TRAM2-OE overexpressed proteins and TRAM2-KO1 and –KO2 downregulated proteins. We have detailed the cutoffs used in this analysis in the figure legend (Figure 6D). We still believe that using raw p-values for this purpose is a valid approach, since we are performing the intersection of 4 conditions (each of them with triplicates).

        Reviewer #3: Comments regarding the response to Reviewer #2.

        Many of the comments have been adequately addressed with the exception of the following:

        Comment 1: In Figure 1F.  Comparing reporter activation in MCF7 cells to MCF10A-YAP5SA is not a valid comparison.  While comparison to MCF10A would have been better, using the promotor control (as provided in the main paper figure) would normally be acceptable.

We agree with the reviewer that comparing reporter activities from MCF10A-YAP to MCF7 is not ideal. In fact, reporter assays for enhancers cannot capture the endogenous complexity of enhancer-promoter interaction, and thus are inferior to other methods to measure enhancer activities, such as chromatin marks, Gro-seq (eRNA expression), ChIP-seq, and gene editing of the endogenous regulatory DNA elements. Since this manuscript includes a lot of data obtained from Gro-seq, ChIP-seq and CRISPR KOs of selected regulatory DNA elements in cells (i.e. MCF10A, MCF10A-YAP, MDA-MB-231, and MCF7 cells; e.g. Figure 1E-1F, 2A, 3A, 3D, S2B, Table S3 etc), we do believe that these make the data of the reporter assays redundant and less relevant. Thus, we removed most of the luciferase reporter assay data of the various enhancers from the manuscript (old Figure 1F), making the manuscript more focused and accurate, without affecting any of the conclusions and findings.

        However, there is a caveat regarding the reporter construct. 

        The authors indicate they subcloned the enhancer downstream of the luciferase reporter gene, presumably either up or downstream of the poly-A tail region, rather than downstream of the promoter (ie upstream of the luciferase gene) as I would have expected.  Whether this enhancer placement is considered appropriate needs be confirmed by an expert.  If what was meant was that the enhancer is downstream of the promoter (but upstream of the reporter) then an equivalent ‘control’ DNA fragment should have been used in the control construct, as the distance of the promoter from gene start can significantly impact transcription.  If this is not available/not tested, then only alternative, is to directly compare parental and YAP5SA constructs in the SAME cell line (ie refer to point above).

We cloned our enhancer regions downstream of the luciferase reporter gene (downstream of the PAS). This is a common practice in the field of “regulatory DNA elements” to distinguish enhancer activity from promoter function. See for example our paper in Nature Biotechnology 2016 (Korkmaz et al.,), but also many other ones.

        Comment 3: Regarding using a population rather than clones of the Crispr KO:  the rationale presented by the authors that a pool is better than clones would be true in cases of efficient knockdown, but in contrast to their claim that only a few cells in the pool do not display KO, the blots show a reduction of only 40-60% of control protein levels, suggesting about half the cells do not have a KO.  This heterogeneity (ie an estimated 50% of cells in the pool display KO), must be mentioned in the text.

We appreciate this comment. As suggested by the reviewer, we mentioned the KO efficiency in the text. Please see the sentence “CRISPR-mediated targeting of TRAM2 indicated approximately 50% TRAM2 knockout by DNA mutation and western blotting analyses (Figure 3F, 3H and Figure S5C).” in page11.

        Considering this KO efficiency, I was initially puzzled to see that the authors show almost complete inhibition of growth (Fig 3E) in the heterogeneous population.  However, it became apparent that a truncated Y-axis was used (ie it did not start at zero), creating a misleading impression of the results.  This should be corrected in this and all other plots.

We appreciate this comment. We followed the comment and corrected the Y-axis for all the figures related to growth experiments (start from zero now).

        Comment 5: Regarding, the request to confirm that there is a reduction of FSTL1 protein but not mRNA levels (Fig S7D).  First, I disagree with the author’s response that large scale experiments (Mass Spec and RNAseq) are sufficient for demonstrating differential expression.  The authors provide a panel to address this, but the reduction in FSTL1 protein is extremely modest and while the mRNA does not show any change, the authors neglected to confirm the KO efficiency in the same sample, which presumably is using a pool as above).  Since pools can change depending on culture times, the absence of this control is particularly problematic.

We appreciate this comment. We performed the WB again to measure the TRAM2 and FSTL1 with the same sample this time and replaced the Figure S7D. We observed moderate reduction of TRAM2 (around 40-50%) and also a clear reduction of FSTL1(40-60%).

Comment 5 (continued).  The authors response to the comment “Moreover, the reviewer feels that the significance of FSTL1 as a downstream target of TRAM2/YAP is inadequately represented. It would be meaningful to see whether FSTL1 KO can alleviate the cancer-associated phenotypes shown in Figure 6E-G in YAP-overexpressing cells, or that overexpression of FSTL1 itself is sufficient to accelerate these phenotypes.” is that this is beyond the scope of the manuscript.

We appreciate this comment. We think this is beyond the scope of the paper since many publications already showed the role of FSTL1 (KO or overexpression) in cancer associated phenotypes. We also removed the sentence “Mechanistically, we identified FSTL-1 as a major direct client of TRAM2 that is involved in these phenotypes” from our abstract as suggested by the reviewer in the next comment.

        While, I agree with the authors that so many experiments to support the role of FSTL1 are not required, the abstract states  “Mechanistically, we identified FSTL-1 as a major direct client of TRAM2 that is involved in these phenotypes.” Thus, data supporting this statement is required or the statement must be modified.  There is only one figure that addresses this comment, Fig. 6G on migration, but the control line, ie non-TRAM2 expressing cells, was not included so there is no evidence to support a mechanistic link between TRAM2 and FSTL1, rather it can only be concluded that FSTL1 can control migration.

We appreciate this comment. We removed the sentence “Mechanistically, we identified FSTL-1 as a major direct client of TRAM2 that is involved in these phenotypes” from our abstract as suggested.

 Comments regarding the response to Reviewer #3.

        The reviewer commends the authors for addressing the majority of questions. However, the following issues remain outstanding.  The following suggestions are recommended to improve the final version of the manuscript.

        The request to provide data linking YAP and TRAM2 in cancer cell lines with active YAP rather than only in the YAP mutant engineered line was not provided (ie such as YAP KD or overexpression on TRAM2 mRNA/protein levels). While the authors state “We already validated several key findings with MDA-231 cell line”, it was not indicated where this could be found.  I was unable to locate this data with the exception of the proliferation data in Fig. S6F, which shows a marginal effect.  It should be noted that results derived from an analysis of screens without subsequent validation is not satisfactory.

We appreciate this comment. We are sorry that we did not make it clear in the last round of the revision when we answered this question. We stated that “We already validated several key findings with MDA-231 cell line” and the related results are:

Figure 5B: TRAM2 expression stimulates cell migration in MDA-231 cells.

Figure S5D: Enhancer^TRAM2^-KOs and TRAM2-KOs both affect cell proliferation in MDA-231 cells.

Figure S6C-S6D: proteomic data indicates that EMT signature was downregulated upon TRAM2-KOs in MDA-231 cells which is in line with other results in MCF10A-YAP cells. Figure S6F: TRAM2-KOs slightly affect the cell migration in MDA-231 cells.

Additionally. We performed the Verteporfin treatment using MDA-231 cells and we observed a clear and significant reduction of TRAM2 expression in Verteporfin treated cells. We added this data to supplementary Figure S5B.

        Figure S1: There is no to very moderate increase in YAP expression level in MCF10A-5SA cells vs control (Figure S1A). The authors may want to consider adding YAP localization by IF to validate the point that the observed phenotypic effect is due to the nuclear localization of YAP5SA and not dependent on the total protein level.

We appreciate this comment. We showed the activity of YAP^5SA^ using reporter assays (Figure 6A, 6B). The fact that there is no high over-expression is an advantage not a problem. When we carefully quantified the WB and there is mild overexpression as below. We mentioned this in the main text. Please see the sentence” The mild overexpression of YAP^5SA^ and YAP^5SA-S94A^ were confirmed (Figure S1A)” in page 7.

However, we also performed the IF to measure the YAP signal from nuclear (representative images and statistical analysis). We observed increased YAP nuclear localization in MCF10A-YAP^5SA^ cells compared to MCF10A-control cells. Please see the figures below.

        Figure 3.  The author's response to the reviewer's comments are unsatisfactory.
        I strongly disagree that RNA-seq data does not have to be confirmed.  It would be simple to check TRAM2 mRNA levels by qPCR.  This is much more quantitative than immunoblotting.
        The conclusions regarding the effect of eTRAM2 KO on cell proliferation, would have been substantially stronger if the authors used multiple verified clones rather than a heterogeneous pool of cells.  However, this issue alone would not preclude publication.

We appreciate this comment. We performed qPCRs to measure TRAM2 levels upon enhancer-Kos and observed around 50% reduction of TRAM2 mRNA level which is in line with our RNAseq data. We added this result to supplementary Figure S4C.

        Figure 5. The TRAM2 expression blot is overexposed.  The authors should determine if there is a difference in the TRAM2 protein levels with a lower amount of protein lysate to overcome the saturation issue.

We appreciate this comment. However, we never over-exposed the blots during their development. We have applied a color LUT (look up table) to the original images corresponding to Figures S6A and S6B in our manuscript. This LUT shows pixels with a value of 0 (pure black, overexposed in this case) in magenta and pixels with a value of 255 (pure white, underexposed in this case) in blue. As you can see in the attached figure bellow, the original raw images of TRAM2 (left part) were not overexposed (there are only few pixels in magenta). We agree though that the final figures in our previous manuscript (right part of attached figure bellow) are slightly saturated, due to excessive brightness/contrast to enhance visibility. Now, we have assembled the figures again using a more moderate brightness/contrast adjustment (Figure S6A, S6B).

        Figure 6. In the new data Figure S7D, there is a very moderate effect of TRAM2-KO in MCF10A-YAP5SA cells on FSTL-1 protein level.  The authors should consider either validating by using single clones or determining the impact of TRAM2 Knockout/knockdown on FSTL-1 using a cancer cell line with active YAP such as MDA-MB231 cells.

We appreciate this comment. We measured TRAM2 and FSTL1 expression upon TRAM2-KOs in MDA-231 cells and we observed around 40-50% TRAM2 KO and clear reduction (70-80%) of FSTL1 expression. We now add this result to supplementary Figure S7E.

**Third round of review**

**Reviewer 1**

The response is appropriate.

**Reviewer 3**

Comments sufficiently addressed.
